# Mitochondrial Transplantation Suppresses mtDNA-cGAS/STING-Mediated Innate Immunity by Enhancing PINK1/Parkin-Dependent Mitophagy to Attenuate Keloid Fibrosis

**DOI:** 10.3390/antiox15091120

**Published:** 2026-09-04

**Authors:** Wenjing Wang, Yuanbo Liu, Jipeng Song, Zouzou Yu, Zixiang Chen, Hu Jiao

**Affiliations:** 1 Department of Plastic and Reconstructive Surgery, Plastic Surgery Hospital, Chinese Academy of Medical Sciences and Peking Union Medical College, Beijing 100144, China; b2024004024@student.pumc.edu.cn (W.W.); yb5886@163.com (Y.L.); songjp19800357520@163.com (J.S.); pumcyu@126.com (Z.Y.); chenzixiangcc@163.com (Z.C.); 2 Department of Aesthetic Medicine, Beijing Jishuitan Hospital, Capital Medical University, Beijing 102208, China

**Keywords:** keloids, mitophagy, mitochondrial transplantation, cGAS/STING signaling, innate immunity, adipose-derived stem cells, fibrosis

## Abstract

Keloids are characterized by fibrosis and chronic inflammation, but links between mitochondrial dysfunction and keloid pathogenesis remain unclear. This study examined whether impaired PINK1/Parkin-dependent mitophagy is associated with mitochondrial DNA (mtDNA)-mediated innate immune activation and fibrosis in keloids, and evaluated mitochondrial transplantation as a potential therapeutic strategy. Primary keloid fibroblasts (KFs), normal skin fibroblasts (NFs), adipose-derived stem cells (ADSCs), human keloid tissues, and human keloid xenografts in immunodeficient BALB/c nude mice were analyzed using ultrastructural, molecular, and functional approaches. Freshly isolated NF-derived mitochondria (nMito) and ADSC-derived mitochondria (aMito) were compared at protein-equivalent doses. KFs exhibited mitochondrial abnormalities, impaired oxidative phosphorylation, increased reactive oxygen species, mtDNA leakage, and cGAS/STING pathway activation. Elevated PINK1 expression, reduced Parkin expression and p62 accumulation were consistent with impaired downstream mitophagic clearance. Both nMito and aMito were associated with improved mitochondrial function, changes in mitophagy-related markers, reduced cytosolic mtDNA and cGAS/STING signaling, and attenuated fibroblast activation, with greater aMito-associated changes in selected endpoints. In xenografts, intralesional administration of either mitochondria improved collagen organization and reduced fibrotic and inflammatory signaling. Together, these findings link altered PINK1/Parkin-dependent mitophagy to mtDNA-driven inflammation and fibrosis and support mitochondrial transplantation as a potential organelle-based therapeutic approach.

## 1. Introduction

Keloids are fibroproliferative skin disorders characterized by excessive extracellular matrix (ECM) deposition, aberrant dermal fibroblast activation, and persistent inflammatory cell infiltration [1]. Unlike physiological wound healing, keloids extend beyond the boundaries of the original injury and rarely regress spontaneously [2]. Patients frequently experience pain, itching, aesthetic disfigurement, and, in severe cases, functional impairment, substantially compromising quality of life [3]. Despite the availability of multiple therapeutic approaches (e.g., surgical excision, radiotherapy, corticosteroid injections), treatment outcomes remain suboptimal with high recurrence rates, underscoring an unmet need for mechanism-driven therapeutic strategies [4]. Studies have increasingly suggested that keloids are not merely disorders of excessive collagen deposition, but rather complex pathological scars characterized by metabolic dysregulation and chronic inflammatory activation [5,6]. Mitochondria serve as central regulators of cellular metabolism, stress adaptation, and immune signaling; accordingly, maintenance of mitochondrial homeostasis is essential for cellular metabolic adaptation and stress responses [7]. Emerging evidence positions mitochondrial homeostasis disruption at the intersection of metabolic dysregulation and innate immune activation in fibrotic disease [8].

As central regulators of cellular redox homeostasis, mitochondria are closely involved in oxidative stress responses and are vulnerable to redox imbalance [9]. In response to mitochondrial stress, damaged mitochondria are selectively eliminated through mitophagy to preserve mitochondrial quality control and homeostasis [10]. Defective mitophagy leads to the accumulation of damaged mitochondria, progressive deterioration of oxidative phosphorylation (OXPHOS), excessive reactive oxygen species (ROS) production, and mitochondrial DNA (mtDNA) leakage, thereby driving cellular dysfunction [10]. Recent studies have shown that keloid fibroblasts (KFs) exhibit disrupted mitochondrial homeostasis, as evidenced by the accumulation of dysfunctional mitochondria [11]. The PTEN-induced putative kinase 1 (PINK1)/Parkin axis plays a pivotal role in mitochondrial quality control by initiating the selective removal of damaged mitochondria through mitophagy [12]. However, reduced Parkin expression has been reported in keloids, suggesting that defective PINK1/Parkin-dependent mitophagy may contribute to mitochondrial homeostasis disruption during keloid progression [13].

Accumulating evidence indicates that dysfunctional mitochondria actively participate in innate immune activation through mtDNA-mediated inflammatory signaling [14]. Cytosolic mtDNA released from damaged mitochondria functions as a damage-associated molecular pattern (DAMP) and is recognized by cyclic GMP-AMP synthase (cGAS), leading to activation of the stimulator of interferon genes (STING) pathway and downstream inflammatory cascades [15]. In multiple fibrotic disorders, such as hepatic fibrosis, aberrant activation of the mtDNA-cGAS/STING axis drives fibrosis by activating innate immunity, thereby promoting fibroblast activation and pathological ECM remodeling [16]. In keloids, chronic inflammatory activation is a well-recognized pathological feature [1]. Importantly, activation of cGAS/STING signaling further induces downstream NF-κB signaling and enhances the secretion of pro-inflammatory and pro-fibrotic mediators (e.g., CCL-2, IL-6, TGF-β1), all of which are closely associated with macrophage recruitment, fibroblast activation, and excessive collagen deposition [17]. These observations raise the possibility that defective mitophagy may cause cytosolic mtDNA accumulation, which in turn engages the cGAS/STING innate immunity pathway within the keloid stromal–immune niche.

Mitochondrial transplantation has recently emerged as a promising therapeutic strategy for restoring mitochondrial homeostasis [18]. Transplanted exogenous mitochondria can integrate into recipient cells, replenish the functional mitochondrial pool, normalize cellular bioenergetics, and attenuate oxidative stress. In addition to metabolic restoration, mitochondrial transplantation has been shown to facilitate mitochondrial quality control by promoting mitophagy and clearance of damaged mitochondria [19]. Mitochondrial transplantation has demonstrated therapeutic potential in several diseases (e.g., pulmonary fibrosis, hepatic injury, cardiac ischemia–reperfusion injury, neurodegenerative disorders) [20]. Among available donor cell sources, adipose-derived stem cells (ADSCs) possess several practical advantages, including abundant accessibility and minimally invasive harvesting [21]. Nevertheless, mitochondria have been shown to exhibit tissue-specific functional heterogeneity, and metabolic compatibility between donor mitochondria and recipient cells has been identified as a crucial determinant of transplantation efficacy, highlighting the importance of optimizing donor cell selection to achieve superior therapeutic outcomes in cutaneous fibrotic diseases [22]. To date, mitochondrial transplantation has not been investigated as a therapeutic strategy for keloids.

In the present study, we systematically characterized mitochondrial dysfunction, mitophagy impairment, and mtDNA-cGAS/STING-mediated innate immune activation in keloids, and evaluated mitochondrial transplantation as a therapeutic strategy to restore mitochondrial homeostasis, thereby suppressing cGAS/STING-mediated innate immune signaling. Furthermore, given the emerging recognition of donor-dependent mitochondrial heterogeneity, we compared the effects of ADSC-derived mitochondria (aMito) and normal skin fibroblast-derived mitochondria (nMito) administered at the same protein-equivalent dose. We observed that mitochondrial transplantation was accompanied by improved mitochondrial function, changes in PINK1/Parkin-related mitophagy markers, reduced cytosolic mtDNA accumulation and cGAS/STING signaling activation, and the attenuation of fibroblast activation and stromal inflammatory remodeling in keloids. Collectively, our findings identify mitochondrial homeostasis disruption as a hallmark of keloid pathogenesis and provide preclinical proof-of-concept for mitochondrial transplantation as an organelle-targeted immunometabolic therapy for cutaneous fibrosis.

## 2. Materials and Methods

### 2.1. Patients and Clinical Sample Collection

Keloid tissue specimens were obtained from patients undergoing surgical excision. Inclusion criteria were as follows: (1) clinical and histopathological diagnosis of keloid; (2) lesion duration of at least 12 months; and (3) no receipt of any local or systemic treatment within 6 months prior to enrollment. Patients with a history of systemic autoimmune diseases, chronic dermatopathies, metabolic disorders, or malignant neoplasms were excluded. Normal skin specimens were obtained from patients undergoing abdominoplasty, and adipose tissue was collected from patients undergoing liposuction. All tissue specimens were collected at our facility. The study was conducted in accordance with the Declaration of Helsinki and approved by the Institutional Review Board. The demographic and clinical characteristics of the human tissue donors are summarized in Supplementary Table S1. Upon collection, each specimen was processed as needed for downstream analyses: one portion was fixed in 2.5% glutaraldehyde for transmission electron microscopy (TEM); one portion was fixed with 4% paraformaldehyde (PFA) for histological analyses; one portion was snap-frozen and stored at −80 °C for molecular analyses; and one portion was used for primary cell isolation or in vivo implantation.

### 2.2. Cell Isolation and Culture

Primary KFs and normal skin fibroblasts (NFs) were isolated using the explant culture method, as previously described [23]. Briefly, specimens were trimmed of subcutaneous fat under sterile conditions, minced into pieces of approximately 1 mm^3^, and washed six times with PBS (PB180327; Pricella, Wuhan, China) containing 100 U/mL penicillin and 100 µg/mL streptomycin. The pieces were transferred to 100 mm culture dishes and allowed to air-dry for 20 min in a laminar-flow cabinet to facilitate adhesion. Dulbecco’s Modified Eagle’s Medium (DMEM; 11885084; Gibco, Grand Island, NY, USA) supplemented with 10% fetal bovine serum (FBS; 10099141C; Gibco), 100 U/mL penicillin, and 100 µg/mL streptomycin was added. To avoid disturbing early tissue adhesion, the medium was not replaced during the first week. Thereafter, the medium was replaced every 3 days. Fibroblasts migrating from the explants were passaged upon reaching 80–90% confluence using 0.25% trypsin/0.02% EDTA (25200056; Gibco). Cells at passages 2–3 (P2–P3) were used for the following experiments.

ADSCs were isolated from lipoaspirate specimens by enzymatic digestion, as previously described [21]. Briefly, the aspirate was washed three times with equal volumes of PBS to remove erythrocytes and debris. The washed tissue was incubated with 0.2% collagenase type I (C0130; Sigma-Aldrich, St. Louis, MO, USA) in PBS on a constant temperature shaker set at 37 °C and 100 rpm for 50 min. Digestion was then terminated by adding an equal volume of complete medium. The digested suspension was filtered through a 100 µm cell strainer, centrifuged at 300× *g* for 5 min at room temperature, and the pelleted stromal vascular fraction was resuspended and plated in T75 flasks in complete ADSC medium (DMEM supplemented with 10% FBS, 100 U/mL penicillin, and 100 µg/mL streptomycin). Non-adherent cells were removed after 48 h by medium replacement. ADSCs between P3 and P5 were employed in this study. ADSC identity was confirmed by flow cytometric immunophenotyping at P3. Cells were incubated with fluorochrome-conjugated antibodies against CD105, CD90, CD73, CD45, CD14, CD34, CD19, and HLA-DR (all from Proteintech, Wuhan, China) at 1 μL per 10^6^ cells for 20 min at room temperature in the dark. Isotype-matched antibody controls were included. Data were acquired on a BD FACSCanto II flow cytometer (Becton Dickinson, Franklin Lakes, NJ, USA) and analyzed with FlowJo v10 (BD Biosciences, Ashland, OR, USA). In accordance with the International Society for Cell Therapy (ISCT) criteria, ADSCs were defined as cells expressing ≥95% of CD105, CD90, and CD73 and <2% of CD45, CD14, CD34, CD19, and HLA-DR. Multilineage differentiation capacity was assessed using the Human Adipogenic Differentiation Induction Kit (HUXMD-90031; Cyagen Biosciences, Guangzhou, China) and the Human Osteogenic Differentiation Induction Kit (HUXMD-90021; Cyagen Biosciences), following the manufacturer’s protocols. Adipogenic differentiation was confirmed by Oil Red O staining and osteogenic differentiation was confirmed by Alizarin Red S staining.

The human monocytic cell line THP-1 (CL-0233; Procell Life Science & Technology, Wuhan, China) was cultured and differentiated into resting macrophages (M0) as previously described [24]. Briefly, cells were maintained in RPMI-1640 medium (11875093; Gibco) supplemented with 10% FBS and 1% penicillin/streptomycin and differentiated by treatment with 80 ng/mL PMA (P8139; Sigma-Aldrich) for 24 h followed by a 24 h rest period in fresh medium. All cells were maintained at 37 °C in a humidified atmosphere of 5% CO_2_.

### 2.3. Pharmacological Interventions

H-151 (S6652; Selleck, Houston, TX, USA) is a covalent and selective small-molecule antagonist of STING. H-151 was applied to KFs at 1 µmol/L for 24 h, a concentration chosen based on previous reports [25]. H_2_O_2_ (88597; Sigma-Aldrich) was used to promote cytosolic mtDNA release. KFs were exposed to 6 mmol/L H_2_O_2_ for 1 h to examine the subsequent activation of the cGAS/STING pathway [26]. To assess cell viability, KFs were exposed to 0, 0.5, 1, 2, 4, or 6 mmol/L H_2_O_2_ for 1 h. After treatment, cells were washed twice with PBS and incubated with fresh medium containing 10% Cell Counting Kit-8 (CCK-8; C0038; Beyotime, Shanghai, China) reagent for 1 h at 37 °C. Absorbance was measured at 450 nm. To evaluate the contribution of fibroblast-derived cytokines, neutralizing antibodies against human IL-6 (AF-206-NA; R&D Systems, Minneapolis, MN, USA) and CCL-2 (AF-279-NA; R&D Systems) were added to the co-culture medium at final concentrations of 1 μg/mL and 5 μg/mL, respectively.

### 2.4. Gene Silencing

For genetic knockdown of Parkin, NFs were transfected with siRNA targeting *PRKN* (the gene encoding Parkin; sc-42158; Santa Cruz Biotechnology, Dallas, TX, USA) or a scrambled non-targeting control siRNA. Transfection was executed using siRNA Transfection Reagent (sc-29528; Santa Cruz Biotechnology) and siRNA Transfection Medium (sc-36868; Santa Cruz Biotechnology) according to the manufacturer’s optimized protocols. Briefly, NFs were seeded in 6-well plates and cultured to 60–80% confluence. The siRNA duplexes and transfection reagents were diluted separately, incubated at room temperature for 30 min to form complexes, and then gently added to the cells. After 6 h of incubation at 37 °C, the medium was replaced with complete DMEM containing 10% FBS. At 48 to 72 h post-transfection, cells were harvested, and the knockdown efficiency of Parkin was validated via Western blot analysis.

### 2.5. Keloid Xenograft Model

This animal study was designed, conducted, and reported in accordance with the ARRIVE 2.0 guidelines. All experimental procedures were approved by the Medical Ethics Committee and performed in compliance with the NIH Guide for the Care and Use of Laboratory Animals. Six-week-old female BALB/c nude mice (Beijing Weitong Lihua Experimental Animal Technology Co., Ltd., Beijing, China) were housed under specific-pathogen-free conditions with a 12 h light/dark cycle, controlled temperature (22 ± 2 °C), and ad libitum access to sterilized food and water. Animals were monitored daily for general health status, body weight, activity, grooming behavior, wound healing, and signs of pain or distress. Humane endpoints were predefined before study initiation. No unexpected deaths or premature exclusions occurred during the study. Only female mice were included in this study; therefore, the animal experiment was not designed to evaluate sex-dependent differences.

After one week of acclimatization, mice were anesthetized with isoflurane (2% for induction, 1% for maintenance) delivered via a nose cone. Fresh keloid tissue specimens were cut into uniform pieces of 5 mm × 5 mm × 3 mm under sterile conditions. Two fragments per mouse were implanted subcutaneously on the dorsum through small incisions, as previously described [27]. Incisions were closed with 6-0 nylon sutures, and sutures were removed at day 7 post-implantation. One week after suture removal (day 14 post-implantation), mice were randomly assigned to the following treatment groups using a computer-generated randomization schedule: Group A, vehicle; Group B, nMito; Group C, aMito. The vehicle group received 50 μL PBS per lesion, whereas the nMito and aMito groups received mitochondria suspended in a final volume of 50 μL PBS. All injections were administered by multipoint intrafocal injection using a 26-gauge needle twice weekly for 2 weeks. Following completion of treatment, the mice were observed for an additional 2 weeks and euthanized (day 42 post-implantation). Six mice per group were used for quantitative analyses. An additional cohort of mice (*n* = 2 per time point) receiving intrafocal injection of fluorescence-labeled mitochondria was used for qualitative analysis. In vivo fluorescence imaging and tissue harvesting were performed at days 1, 3, 5, and 7 post-injection. Cryosections were subsequently prepared and imaged using a Leica TCS SP8 laser scanning confocal microscope (Leica Microsystems, Wetzlar, Germany). Sample size was determined based on previous studies employing similar keloid animal models and on the 3R principles.

Investigators responsible for histological quantification, fluorescence imaging, and molecular analyses were blinded to group allocation during data acquisition and analysis. No animals or data points were excluded from statistical analyses unless predefined humane endpoints were reached. At the end of the study, mice were euthanized by CO_2_ asphyxiation followed by cervical dislocation, in accordance with AVMA 2020 guidelines. The xenografts were carefully excised, cleared of visibly adherent surrounding tissue, and photographed under standardized conditions alongside a metric scale. The implants were then weighed before fixation or snap-freezing. Harvested implants were divided into two portions: one fixed in 4% PFA for histological analyses and one snap-frozen at −80 °C for molecular analyses.

### 2.6. Mitochondria Isolation, Quantification, and Labeling

NF cultures used for nMito preparation were established from 6 independent normal-skin donors, whereas ADSC cultures used for aMito preparation were established from 6 independent lipoaspirate donors. Mitochondria from NFs and ADSCs were isolated using the Mitochondrial Isolation and Protein Extraction Kit (PK10016; Proteintech) according to the manufacturer’s instructions. Briefly, 2 × 10^7^ cells were harvested, washed with ice-cold PBS, and homogenized in mitochondrial isolation buffer on ice. Following centrifugation at 600× *g* for 10 min at 4 °C to remove intact cells and nuclei, the supernatant was further centrifuged at 10,000× *g* for 10 min at 4 °C to obtain crude mitochondria. High-purity mitochondria were subsequently isolated by density-gradient centrifugation. Freshly isolated mitochondria were used within 2 h for subsequent experiments. Mitochondrial protein concentration was determined using a BCA Protein Assay Kit (P0010S; Beyotime) and was used to standardize the administered protein-equivalent dose. Protein normalization was not considered equivalent to normalization of mitochondria number or bioenergetic competence. To evaluate mitochondrial integrity after isolation, donor cells were pre-labeled with MitoTracker Red FM (M22425; Invitrogen, Carlsbad, CA, USA; 200 nM) prior to mitochondrial isolation. Isolated mitochondria were visualized using a confocal microscope. For mitochondrial tracking experiments, NFs and ADSCs were transduced with a mitochondria-targeted mCherry lentiviral construct (pcSLenti-CMV-mito-mCherry; OBiO Technology, Shanghai, China). Isolated mCherry-labeled mitochondria were co-cultured with KFs, and mitochondrial internalization was verified by confocal microscopy.

### 2.7. Mitochondrial Transplantation

For in vitro experiments, isolated mitochondria (25 µg mitochondrial protein per 1 × 10^5^ cells) were added to KFs and centrifuged at 1500× *g* for 5 min at 4 °C to facilitate mitochondrial uptake, as previously described [28]. Cells were then incubated at 37 °C for 24 h, followed by medium replacement before downstream analyses. The mitochondria were derived from individuals other than the corresponding KF or keloid-tissue donor and were therefore considered allogeneic in the human donor–recipient context. For in vivo experiments, exogenous mitochondria were administered at 40 µg per lesion, based on previously reported mitochondrial transplantation studies [29].

### 2.8. TEM Sample Preparation and Imaging

Keloid and normal skin tissue specimens, as well as KF and NF cell pellets, were fixed in 2.5% glutaraldehyde in 0.1 M sodium cacodylate buffer (pH 7.4) at 4 °C for 2 h, post-fixed in 1% osmium tetroxide for 1.5 h at 4 °C, dehydrated through a graded ethanol series, infiltrated with propylene oxide, and embedded in epoxy resin. Ultrathin sections were stained with 2% uranyl acetate and lead citrate, and examined under a transmission electron microscope at an accelerating voltage of 80 kV. Mitochondrial morphology, including cristae integrity, outer membrane continuity, and matrix electron density, was assessed in six random fields per sample.

### 2.9. Mitochondrial Membrane Potential and ROS Intensity Detection

Mitochondrial membrane potential (ΔΨm) and ROS levels were assessed by confocal microscopy and further quantified by flow cytometry. For confocal microscopy, 1 × 10^5^ cells cultured on confocal dishes were stained with JC-1 (C2006; Beyotime) or DCFH-DA (S0033S; Beyotime) according to the manufacturers’ instructions and immediately imaged on a confocal microscope. In polarized mitochondria with high ΔΨm, JC-1 accumulates and forms red-fluorescent J-aggregates; upon mitochondrial depolarization, JC-1 remains in the green-fluorescent monomeric form. DCFH-DA fluorescence was used as a general phenotypic indicator of intracellular oxidant burden associated with oxidative stress. Fluorescence intensities were quantified using Fiji (a distribution of ImageJ2; National Institutes of Health, Bethesda, MD, USA). For flow cytometry, 1 × 10^6^ cells were detached with 0.25% trypsin/EDTA, washed with PBS, and stained with the MitoProbe JC-1 Assay Kit (M34152; Invitrogen) or the Total ROS Detection Kit (88-5930-74; Invitrogen) per the manufacturers’ instructions. The intracellular oxidation-sensitive fluorescence measured using the Total ROS Detection Kit was used as a complementary phenotypic readout rather than as a species- or organelle-specific measurement of ROS. Data were acquired on a BD FACSCanto II flow cytometer and analyzed with FlowJo v10 software.

### 2.10. Metabolic Parameters and Citrate Synthase Activity

Mitochondrial respiratory function was assessed by measuring the oxygen consumption rate (OCR) using the Seahorse XFe96 Extracellular Flux Analyzer (Agilent Technologies, Santa Clara, CA, USA) with the XF Cell Mito Stress Test Kit (103015-100; Agilent Technologies), according to the manufacturer’s protocol. Briefly, KFs were seeded in XFe96 microplates at 1.5 × 10^4^ cells per well 24 h before the assay. Prior to the assay, cells were washed twice with Seahorse XF assay medium and incubated in Seahorse XF DMEM Medium (without phenol red) supplemented with 10 mmol/L glucose, 1 mmol/L sodium pyruvate, and 2 mmol/L L-glutamine (pH 7.4). Cells were equilibrated for 60 min at 37 °C in a non-CO_2_ incubator before measurement. Oligomycin (1.5 μmol/L), FCCP (1.5 μmol/L), and rotenone/antimycin A (0.5 μmol/L each) were sequentially injected to determine basal respiration, maximal respiration, adenosine triphosphate (ATP) production, and spare respiratory capacity. For extracellular acidification rate (ECAR) measurement, cells were washed twice and incubated in Seahorse XF DMEM Medium supplemented with 2 mmol/L L-glutamine (pH 7.4) for 60 min in a non-CO_2_ incubator. Glucose (10 mmol/L), oligomycin (1 μmol/L), and 2-deoxy-D-glucose (2-DG; 50 mmol/L) were sequentially injected to determine glycolysis, glycolytic capacity, and glycolytic reserve. All OCR and ECAR values were normalized to cell number, determined by crystal violet staining in parallel wells. L-lactate levels were measured using the Lactic Acid Assay Kit (BC2235; Solarbio Science & Technology, Beijing, China), and glucose consumption in the conditioned medium was quantified using the Glucose Assay Kit (S0554S; Beyotime), each according to the manufacturer’s instructions. Values were normalized to total protein content as determined by BCA assay.

Citrate synthase activity was measured using a Citrate Synthase Assay Kit (CS0720; Sigma-Aldrich). Cells were washed three times with ice-cold PBS, detached using 0.05% trypsin-EDTA, and collected by centrifugation at 1000× *g* for 5 min at 4 °C. Cell pellets were washed twice with ice-cold PBS to minimize the contribution of extracellularly adherent mitochondria. Whole-cell lysates were prepared using the lysis reagent supplied with the kit and clarified by centrifugation at 12,000× *g* for 10 min at 4 °C. Total protein concentrations were determined using the BCA Protein Assay Kit. Equal amounts of lysate protein were used for all samples. Citrate synthase activity was determined from the rate of formation of 5-thio-2-nitrobenzoic acid following the reaction of free CoA-SH with 5,5′-dithiobis-(2-nitrobenzoic acid). Baseline absorbance at 412 nm was recorded for 2 min before the reaction was initiated by the addition of freshly prepared oxaloacetate. The increase in absorbance at 412 nm was subsequently monitored at 37 °C for 3 min using a microplate reader. The maximum linear rate of change in absorbance was used to calculate citrate synthase activity according to the manufacturer’s instructions.

### 2.11. Western Blot Analysis

Cells and tissues were washed with ice-cold PBS (tissues were minced and homogenized), then lysed in RIPA buffer containing protease and phosphatase inhibitors. Protein concentrations were determined by BCA assay. Equal amounts of protein (20–40 µg) were resolved by SDS-PAGE on 10–12% polyacrylamide gels and transferred onto methanol-activated PVDF membranes. For experiments involving multiple target proteins, equal aliquots of the same whole-cell lysates were loaded in an identical sample order on separate gels and transferred to separate PVDF membranes. Each target protein was detected on a membrane containing all comparison groups from the corresponding experiment. GAPDH was detected on a separately processed membrane loaded with corresponding aliquots of the same lysates and was used as a whole-cell loading reference. Target-band intensities were normalized to the corresponding GAPDH signals from the same experimental set. Membranes were blocked with 5% non-fat dry milk in TBST (50 mmol/L Tris-HCl, 150 mmol/L NaCl, 0.1% Tween-20, pH 7.6) for 1 h at room temperature, except those probed with phospho-specific antibodies, which were blocked with 5% BSA in TBST. Membranes were then incubated overnight at 4 °C with the following primary antibodies: GAPDH (60004-1-Ig; Proteintech; 1:50,000); TOMM20 (AF1717; Beyotime; 1:1000); PINK1 (AF7755; Beyotime; 1:1000); Parkin (14060-1-AP; Proteintech; 1:4000); LC3B (18725-1-AP; Proteintech; 1:1500); p62 (18420-1-AP; Proteintech; 1:10,000); cGAS (26416-1-AP; Proteintech; 1:40,000); STING (19851-1-AP; Proteintech; 1:20,000); phospho-STING (PA5-105674; Invitrogen; 1:1000); TBK1 (ab40676; Abcam, Cambridge, UK; 1:10,000); phospho-TBK1 (AF5959; Beyotime; 1:2000); phospho-NF-κB (80379-2-RR; Proteintech; 1:5000); and TGF-β1 (81746-2-RR; Proteintech; 1:5000). After three washes with TBST, membranes were incubated with HRP-conjugated goat anti-rabbit or goat anti-mouse IgG secondary antibody (Proteintech; 1:5000–1:10,000) for 1 h at room temperature. Bands were visualized by enhanced chemiluminescence and quantified using Fiji.

### 2.12. Mitochondrial DNA Quantification

Total cellular DNA was extracted from cell pellets using the FastPure Cell/Tissue DNA Isolation Mini Kit (DC102-01; Vazyme, Nanjing, China) according to the manufacturer’s protocol. DNA purity and concentration were assessed spectrophotometrically by measuring OD_260_/OD_280_ ratios; samples with ratios between 1.8 and 2.0 were used for downstream analysis. For cytosolic mtDNA quantification, cells were lysed by digitonin-mediated selective plasma membrane permeabilization as previously described [15]. Briefly, cell pellets were resuspended in 250 µL permeabilization buffer (150 mmol/L NaCl, 50 mmol/L HEPES pH 7.4, 25 µg/mL digitonin) and incubated for 10 min at room temperature. The lysate was centrifuged at 1000× *g* for 10 min at 4 °C to pellet intact cells and nuclei. The post-nuclear supernatant was further centrifuged at 17,000× *g* for 10 min at 4 °C to remove mitochondria and residual cellular debris. The resulting supernatant, representing the mitochondria-depleted cytosolic fraction, was processed for DNA isolation, with elution volumes matched to those of the corresponding total DNA samples to ensure equivalent normalization. Quantitative PCR (qPCR) was performed using the 2× ChamQ SYBR qPCR Master Mix (Q311-03; Vazyme) on a CFX96 real-time PCR system (Bio-Rad Laboratories, Hercules, CA, USA). Total mtDNA copy number was assessed using primers targeting *MT-ND1* and *MT-ND2*. Cytosolic mtDNA was quantified using the same primers; successful exclusion of nuclear DNA contamination was confirmed by the absence of amplification signals for the nuclear markers *18SRRNA* and *L1ORF1*. Primer sequences are listed in Supplementary Table S2. To visualize cytosolic mtDNA leakage, cells grown on confocal dishes were first incubated with MitoTracker Deep Red FM (M22426; Invitrogen) at 37 °C for 30 min in the dark to label mitochondria. After washing, cells were then stained with PicoGreen (P2023; UE, Shanghai, China) for 15 min in the dark to label double-stranded DNA. Following three additional washes with PBS, cells were immediately imaged on a confocal microscope. Cytosolic mtDNA was identified as PicoGreen-positive DNA puncta located outside the MitoTracker-labeled mitochondrial network, as previously described [16].

### 2.13. cGAS-mtDNA Immunoprecipitation Assay

The physical interaction between cytosolic mtDNA and cGAS was assessed as previously described [26]. Briefly, cells were crosslinked with 1% formaldehyde for 10 min at room temperature, and the reaction was quenched with glycine. Cells were then gently lysed in hypotonic buffer containing protease inhibitors, followed by 0.2% NP-40 to selectively disrupt the plasma membrane while leaving mitochondria intact. The lysates were centrifuged to remove nuclei and intact mitochondria, and the resulting supernatants were collected as cytosolic fractions. Cytosolic DNA–protein complexes were fragmented by sonication (6 × 10 s pulses with 30 s intervals on ice). After clearing debris by centrifugation, aliquots of the supernatant were saved as input controls. The remaining lysates were incubated overnight at 4 °C with anti-cGAS antibody or normal rabbit IgG (negative control). Immune complexes were captured using Protein G magnetic beads for 2 h at 4 °C, then washed sequentially with low-salt and high-salt buffers. Crosslinks were reversed by incubation at 65 °C for 2 h in the presence of proteinase K. DNA was purified using spin columns. The presence of cGAS-mtDNA was first assessed by conventional PCR using primers specific for *MT-ND1* and *MT-ND2*, and PCR products were resolved by agarose gel electrophoresis. The enrichment of mtDNA associated with cGAS was further quantified by qPCR and normalized to input.

### 2.14. Cytokine Quantification

Conditioned medium from KFs subjected to various treatments was collected after 24 h of incubation in serum-free DMEM. Concentrations of CCL-2, IL-6, and TGF-β1 were measured using the respective ELISA kits (CCL-2: KE00425; IL-6: KE00385; TGF-β1: KE00002; Proteintech) in accordance with the manufacturers’ instructions. Optical density was read at 450 nm on an EnSpire multimode plate reader (PerkinElmer, Singapore), and concentrations were calculated from standard curves.

### 2.15. Macrophage Polarization Assay

To model the immunomodulatory effects of treated KFs on macrophage phenotype, an indirect co-culture system was established using Transwell inserts, which permit passage of secreted factors but prevent direct cell–cell contact. M0 macrophages were seeded in the lower chamber at 2 × 10^5^ cells per well and allowed to adhere for 12 h. KFs subjected to the designated treatments were seeded in the upper insert compartment. Co-culture was maintained for 48 h. Following co-culture, macrophages were harvested, washed twice with PBS, and resuspended as single-cell suspensions. Cells were incubated with PE-conjugated anti-human CD206 antibody (PE-65155; Proteintech) for 20 min at room temperature in the dark according to the manufacturer’s instructions. After staining, cells were washed with PBS and resuspended in staining buffer for flow cytometric analysis. Data were collected by BD FACSCanto II and analyzed using FlowJo v10.

### 2.16. Confocal Immunofluorescence and Colocalization Analysis

Cells were seeded onto glass coverslips in 24-well plates and allowed to adhere overnight. Following designated treatments, cells were fixed in 4% PFA in PBS for 15 min at room temperature, washed three times with PBST, and permeabilized with 0.5% Triton X-100 in PBS for 5 min at room temperature. After three additional PBST washes, non-specific binding was blocked with 5% normal goat serum in PBS for 30 min at room temperature. Coverslips were incubated overnight at 4 °C with primary antibodies against LAMP1 (sc-20011; Santa Cruz Biotechnology; 1:100) and TOMM20 (11802-1-AP; Proteintech; 1:100), followed by three washes with PBST and 1 h incubation with species-appropriate Alexa Fluor-conjugated secondary antibodies at 37 °C in the dark. Nuclei were counterstained with DAPI. Images were acquired on a confocal microscope.

### 2.17. Fibroblast Functional Assays

Cell proliferative capacity was evaluated using the CCK-8. Cells were seeded at 5 × 10^3^ cells per well in 96-well plates. CCK-8 reagent was added at the designated time points (24, 48, 72, and 96 h), and absorbance was read at 450 nm after 1 h of incubation at 37 °C. Results were normalized to the 0 h baseline to account for differences in initial seeding efficiency. Cell migratory behavior was assessed across two complementary paradigms: collective migration by scratch wound-healing assay and chemotactic migration by Transwell migration assay. For the scratch assay, confluent monolayers in 6-well plates were scratched with a sterile 200 µL pipette tip, washed twice to remove detached cells, and imaged at 0 and 24 h. Wound closure rate was calculated as [(wound width at 0 h − wound width at 24 h)/wound width at 0 h] × 100% using Fiji software. For the Transwell migration assay, cells were serum-starved for 12 h prior to the assay. Inserts (3464; Corning, NY, USA) were pre-wetted with 100 µL serum-free medium at 37 °C for 1 h. Cells (7 × 10^4^ per insert) in 200 µL serum-free DMEM were loaded into the upper compartment, with 500 µL of 20% FBS-containing medium in the lower chamber as a chemoattractant. After 24 h, non-migrated cells on the upper surface were removed with a cotton swab, and migrated cells on the lower surface were stained with crystal violet for 15 min, rinsed, and air-dried. Randomly selected fields were imaged at 200× magnification and counted using Fiji. Invasive capacity was evaluated using Matrigel Invasion Chambers (354480; Corning) according to the manufacturer’s instructions. Briefly, the chambers were equilibrated to room temperature in a sterile tissue culture hood and rehydrated by adding 500 µL of serum-free DMEM to both the upper and lower compartments, followed by incubation at 37 °C for 2 h. KFs were trypsinized, washed, and resuspended in serum-free DMEM. The 7 × 10^4^ cells in 200 µL of serum-free medium were seeded into the upper chamber, while the lower chamber received 500 µL of DMEM supplemented with 10% FBS as a chemoattractant. After 24 h, invaded cells on the lower surface were fixed, stained with crystal violet, and counted as described above. Fibroblast-mediated collagen secretion was evaluated as an indicator of matrix remodeling activity. Type I collagen secretion in conditioned medium was quantified using a Collagen I ELISA Kit (KE00395; Proteintech) per the manufacturer’s protocol, as described above for cytokine ELISA.

### 2.18. Histological, Immunohistochemical, and Immunofluorescence Evaluation

Paraformaldehyde-fixed tissues were dehydrated through a graded alcohol series, cleared in xylene, and embedded in paraffin. Sections of 4 µm thickness were cut on a rotary microtome. Sections were stained with hematoxylin and eosin (H&E) for general morphology, Masson’s trichrome for collagen distribution, and Sirius Red for fibrillar collagen visualization. Immunohistochemistry was performed using an HRP-based detection system following standard antigen retrieval (10 mM sodium citrate buffer, pH 6.0, 95 °C, 20 min). Primary antibodies were applied overnight at 4 °C: TOMM20 (11802-1-AP; Proteintech; 1:200); Parkin (14060-1-AP; Proteintech; 1:200); LC3B (18725-1-AP; Proteintech; 1:200); p62 (18420-1-AP; Proteintech; 1:200); cGAS (26416-1-AP; Proteintech; 1:400); phospho-STING (PA5-105674; Invitrogen; 1:100); and phospho-NF-κB (82335-1-RR; Proteintech; 1:100). Signal was developed with 3,3′-diaminobenzidine (DAB) and counterstained with hematoxylin. Images of prepared sections were acquired with an EasyScan NFC digital slide scanner (Motic, Hong Kong, China). Sirius Red-stained sections were examined under polarized light microscopy. Red-orange/yellow and green birefringent collagen fibre areas were quantified by color-threshold segmentation under consistent image acquisition and analysis settings. For immunofluorescence, paraffin-embedded sections were deparaffinized in xylene, rehydrated through a graded alcohol series, and subjected to heat-induced antigen retrieval in EDTA buffer at 95 °C for 20 min. Endogenous peroxidase activity was quenched with 3% hydrogen peroxide for 15 min at room temperature. Sections were blocked with normal goat serum for 30 min at room temperature and incubated with anti-PINK1 (AF7755; Beyotime; 1:100) overnight at 4 °C. Sections were then incubated with Alexa Fluor-conjugated secondary antibody, counterstained with DAPI, and mounted with anti-fade medium. The slides were imaged using a confocal microscope.

### 2.19. Bulk RNA-Seq and Bioinformatic Analysis

The publicly available GEO dataset GSE145725 was retrieved from the NCBI Gene Expression Omnibus (GEO) database and processed using the limma package in R (version 4.2.1; R Foundation for Statistical Computing, Vienna, Austria) [30]. For mitophagy scoring, the normalized GSE145725 expression matrix was mapped to human gene symbols, and expression values from multiple probes corresponding to the same gene were averaged. Single-sample gene set enrichment analysis, implemented in the GSVA R package, was applied using the GOBP_MITOPHAGY gene set (GO:0000423), obtained from the MSigDB C5 Gene Ontology Biological Process collection [31]. The resulting enrichment score represented the relative expression activity of the mitophagy-related gene set in each sample and was not interpreted as a direct measurement of mitophagic flux. Functional enrichment analyses were performed using the KOBAS platform with Bonferroni correction (adjusted *p* < 0.05). GSEA for key mitochondrial regulators was conducted using the clusterProfiler package in R. Total RNA was extracted from untreated and aMito-treated KFs obtained from three independent KF donors, with one untreated sample and one aMito-treated sample derived from each donor (three donor-matched pairs; *n* = 3 biological replicates per condition), and submitted to Novogene Co., Ltd. (Beijing, China) for sequencing. The nMito condition was not included in this exploratory two-group RNA-seq experiment. RNA quality was assessed using an Agilent 2100 Bioanalyzer. Samples meeting quality thresholds were sequenced (paired-end 150 bp) on the Illumina NovaSeq 6000 platform. Clean reads were quality-filtered with fastp, aligned to the human reference genome (GRCh38) using HISAT2, and quantified using featureCounts. Differential expression was determined using DESeq2 (|log2FC| ≥ 1, adjusted *p* < 0.05). Data visualization was conducted on the Novogene cloud platform (https://magic.novogene.com; accessed on 18 January 2026).

### 2.20. Statistical Analysis

All statistical tests were performed using GraphPad Prism 9.0 (GraphPad Software, San Diego, CA, USA). Prior to the analyses, all data were examined for the normality of residuals and homogeneity of variances. Data are presented as mean ± standard deviation (SD). Normally distributed data were analyzed by two-tailed Student’s *t*-test (two groups) or one-/two-way ANOVA (three or more groups). Non-normally distributed data were analyzed via Mann–Whitney *U* test (two groups) or Kruskal–Wallis test (three or more groups). A two-tailed *p* < 0.05 was considered statistically significant. Statistical significance is denoted as follows: *^, #^ *p* < 0.05; **^, ##^ *p* < 0.01; ***^, ###^ *p* < 0.001; ****^, ####^ *p* < 0.0001. All experiments were performed with a minimum of three independent biological replicates.

## 3. Results

### 3.1. Mitochondrial Structural Abnormalities and Functional Impairment in Keloid Fibroblasts

**Figure 1:**
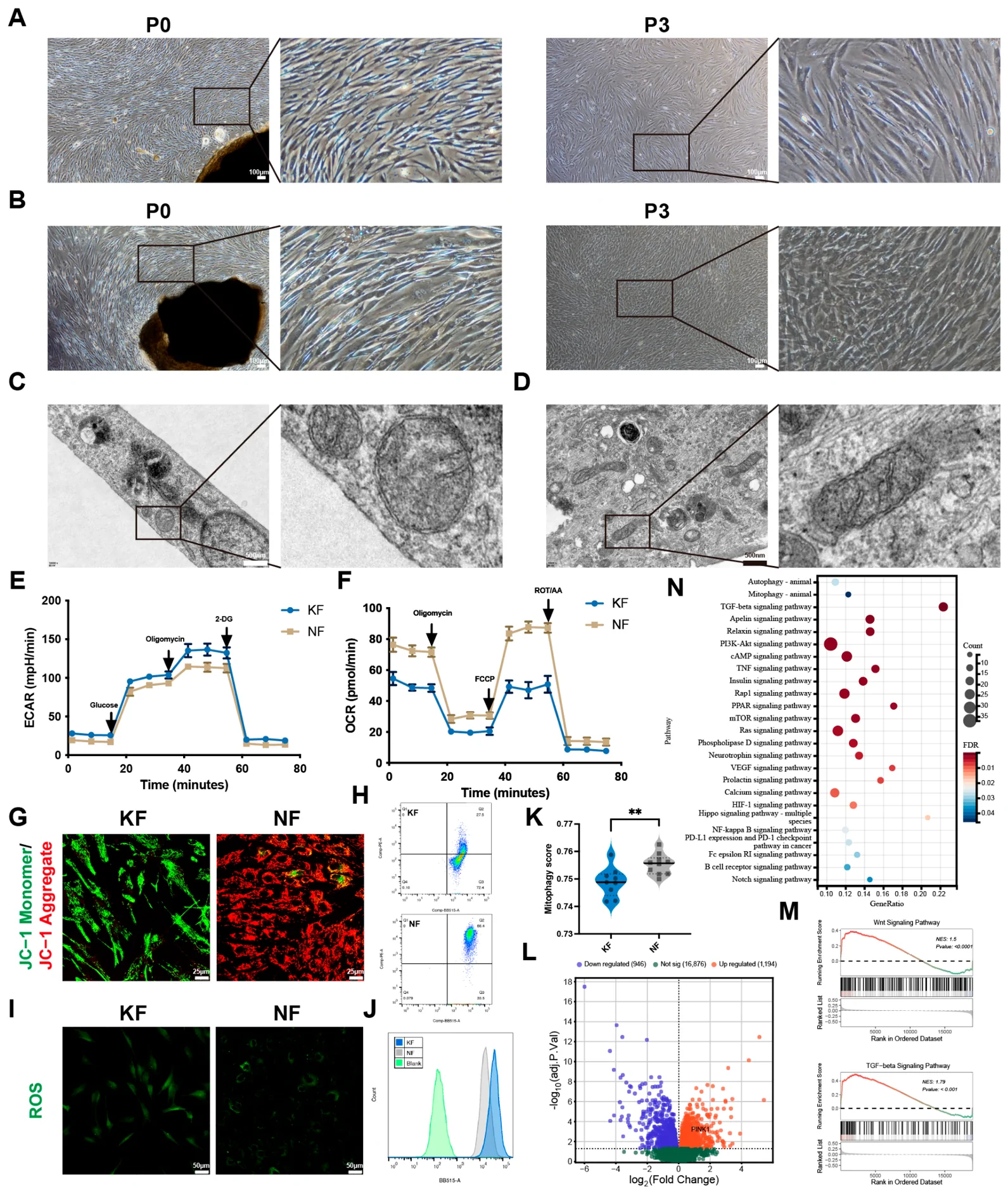
Mitochondrial structural abnormalities and functional impairment in keloid fibroblasts. (**A**,**B**) Representative images of primary fibroblasts isolated from keloid tissues (KFs, (**A**)) and normal skin tissues (NFs, (**B**)). (**C**,**D**) Representative transmission electron microscopy (TEM) images showing mitochondrial ultrastructure in KFs (**C**) and NFs (**D**). (**E**) Representative Seahorse extracellular flux traces of extracellular acidification rate (ECAR) in KFs and NFs under basal conditions and following sequential injection of glucose, oligomycin, and 2-DG. (**F**) Representative Seahorse extracellular flux traces of oxygen consumption rate (OCR) in KFs and NFs. (**G**,**H**) Representative confocal microscopy images (**G**) and flow-cytometric plots (**H**) of JC-1-stained KFs and NFs. In the flow-cytometric analysis, the reported parameter was the percentage of cells classified within the JC-1 aggregate-positive gate on the red-versus-green fluorescence plot, using identical acquisition and gating settings across all groups. (**I**,**J**) Representative confocal microscopy images (**I**) and flow cytometric histograms (**J**) of KFs and NFs showing intracellular ROS levels. (**K**) Comparison of mitophagy scores between KFs and NFs in GSE145725. (**L**) Volcano plot of DEGs identified from the GEO dataset GSE145725. (**M**) GSEA plots showing enrichment of the Wnt and TGF-β signaling pathways correlated with elevated PINK1 expression in GSE145725. (**N**) KEGG pathway enrichment analysis performed using the GSE145725-derived DEG list. Data are presented as mean ± SD from *n* = 3 biological replicates per group. The Seahorse traces were analyzed using two-way repeated-measures ANOVA. Other comparisons between two groups were analyzed using two-tailed Student’s *t*-test. ** *p* < 0.01.

Typical spindle-shaped fibroblasts migrated from both keloid and normal skin tissue explants and exhibited characteristic fibroblastic morphology after culture (Figure 1A,B). To examine whether mitochondrial homeostasis is disrupted in KFs, we first assessed mitochondrial ultrastructure by TEM. Compared with NFs, KFs displayed conspicuous ultrastructural abnormalities, including organelle swelling, extensive cristae disruption and fragmentation, a marked reduction in matrix electron density, and vacuolar degeneration (Figure 1C,D).

Given that mitochondrial ultrastructural damage is closely coupled with bioenergetic dysfunction, we next evaluated mitochondrial metabolic function [32]. Seahorse extracellular flux analysis revealed that KFs displayed elevated ECAR compared with NFs, reflecting a shift toward aerobic glycolysis (Figure 1E). The OCR was markedly reduced in KFs, indicating compromised mitochondrial respiratory activity (Figure 1F). Quantitative analysis further demonstrated reductions in basal respiration, maximal respiration, ATP production, and spare respiratory capacity in KFs (Supplementary Figure S1A). JC-1 staining showed a significantly lower aggregate-positive fraction in KFs than in NFs, as determined by confocal microscopy and flow cytometry (Figure 1G,H and Supplementary Figure S1B,C), indicating mitochondrial membrane depolarization and loss of ΔΨm. Furthermore, ROS levels were elevated in KFs compared with NFs, as assessed by confocal microscopy and flow cytometry (Figure 1I,J and Supplementary Figure S1D,E).

To further validate the involvement of mitochondrial dysfunction in keloid pathogenesis, we performed bioinformatic analyses using the publicly available GEO dataset GSE145725. Gene-set analysis showed that the mitophagy-related enrichment score was lower in KFs than in NFs (Figure 1K). Differential-expression analysis identified differentially expressed genes (DEGs) between KFs and NFs, as summarized in the volcano plot (Figure 1L). Notably, PINK1, a well-established sentinel kinase that accumulates selectively on depolarized and damaged mitochondria, was significantly upregulated in KFs within this dataset (Supplementary Figure S1F), consistent with our experimental observation of mitochondrial membrane depolarization. To elucidate the biological pathways associated with PINK1 dysregulation, GSEA was performed based on PINK1 expression levels. Elevated PINK1 expression was positively associated with several pathways implicated in keloid development, including the Wnt and TGF-β signaling pathways (Figure 1M), suggesting a close relationship between mitochondrial damage and fibrotic activation. KEGG enrichment analysis performed using the GSE145725-derived DEG list identified pathways related to mitophagy, NF-κB signaling, and other inflammatory processes (Figure 1N). Collectively, these findings suggest that KFs exhibit mitochondrial structural abnormalities accompanied by impaired oxidative metabolism, mitochondrial membrane depolarization, and excessive ROS accumulation. Orthogonal bioinformatic analyses of an independent transcriptomic cohort corroborated these experimental observations, further revealing the presence of mitochondrial damage and mitochondrial homeostasis disruption in KFs.

### 3.2. Defective PINK1/Parkin-Dependent Mitophagy in Keloid Tissues and Fibroblasts

**Figure 2:**
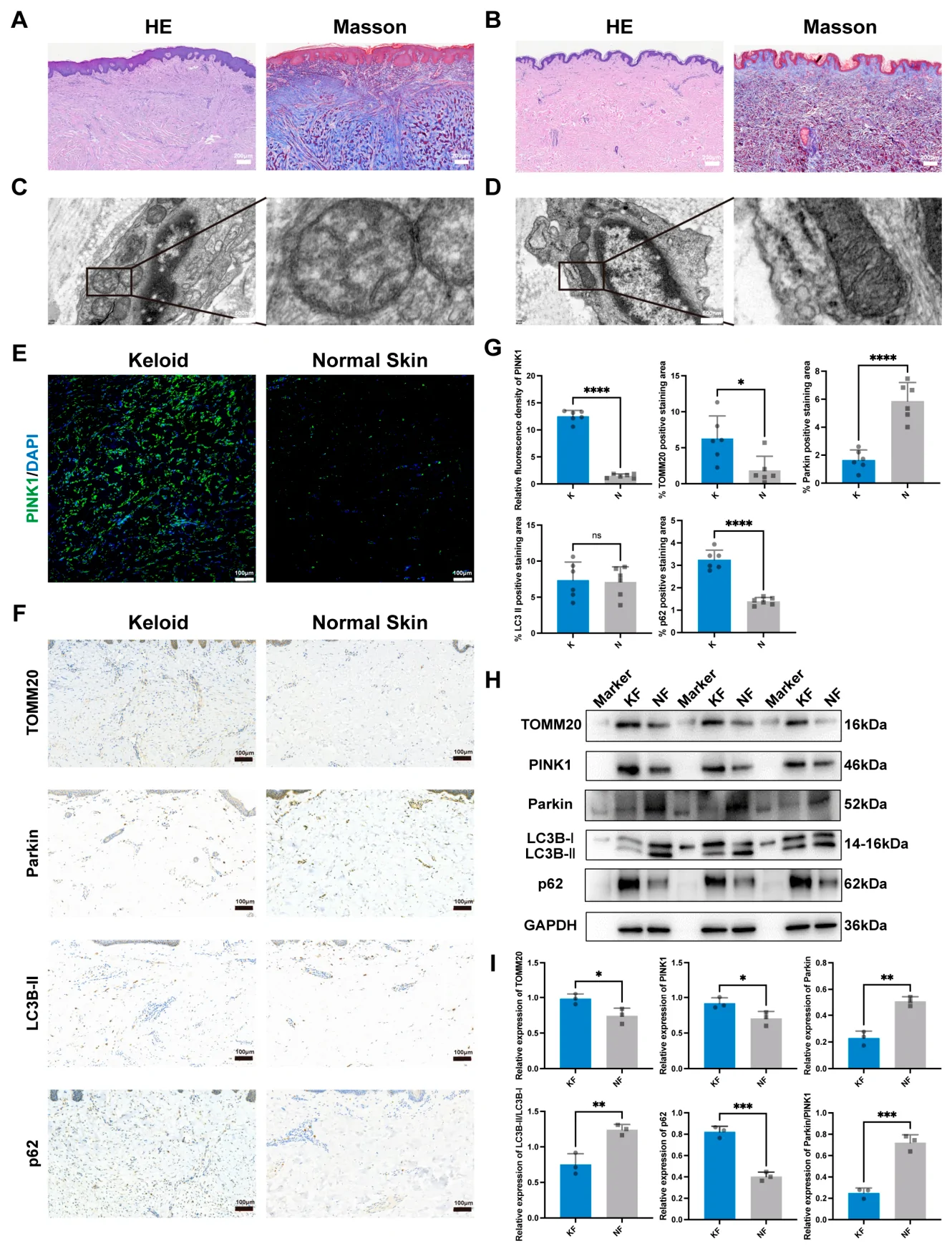
Defective PINK1/Parkin-dependent mitophagy in keloid tissues and fibroblasts. (**A**) Representative H&E and Masson’s trichrome staining of keloid tissue, showing disrupted dermal architecture and dense, disorganized collagen bundles. (**B**) Representative H&E and Masson’s trichrome staining of normal skin, showing relatively preserved dermal architecture and more regularly organized collagen bundles. (**C**) Representative TEM images showing mitochondrial ultrastructure in keloid dermis. (**D**) Representative TEM images showing mitochondrial ultrastructure in normal skin dermis. (**E**) Representative immunofluorescence staining of PINK1 in keloid and normal skin tissues. PINK1 is shown in green and nuclei are counterstained with DAPI in blue. (**F**) Representative immunohistochemical staining showing TOMM20, Parkin, LC3B-II, and p62 protein expression in keloid and normal skin tissues. (**G**) Quantitative analysis of immunofluorescence (PINK1; (**E**)) and immunohistochemical (TOMM20, Parkin, LC3B-II, p62; (**F**)) staining. (**H**) Representative Western blot images showing TOMM20, PINK1, Parkin, LC3B, and p62 expression in KFs and NFs. Full uncropped blot is shown in Supplementary Figure S2. (**I**) Quantitative analysis of protein expression levels shown in (**H**). For tissue-based analyses, *n* = 6 independent biological samples per group; for Western blot analyses, *n* = 3 biological replicates per group. Statistical significance was determined using two-tailed Student’s *t*-test. * *p* < 0.05, ** *p* < 0.01, *** *p* < 0.001, **** *p* < 0.0001.

The accumulation of structurally damaged and functionally impaired mitochondria suggests impaired mitochondrial quality control. Next, we systematically evaluated PINK1/Parkin-dependent mitophagy in keloid tissues and primary fibroblasts. At the tissue level, H&E and Masson’s trichrome staining confirmed the characteristic keloid pathology, including dense disorganized collagen deposition and dermal architectural disruption (Figure 2A,B). TEM of the dermal compartment revealed mitochondria with swelling and cristae fragmentation in keloids, whereas normal skin dermis harbored mitochondria with intact cristae and dense matrix (Figure 2C,D). Immunofluorescence confirmed markedly elevated PINK1 expression in keloid tissues relative to normal skin (Figure 2E), corroborating the bioinformatic findings and further substantiating the in situ accumulation of depolarized mitochondria within the keloid dermis. Immunohistochemical analysis showed increased TOMM20 and p62 expression, reduced Parkin expression, and no significant change in LC3B-II in keloid tissues compared with normal skin tissues (Figure 2F,G). The concurrent accumulation of structurally damaged mitochondria and TOMM20, together with elevated PINK1 expression, reduced Parkin expression, and p62 accumulation, was consistent with defective PINK1/Parkin-dependent mitophagy.

At the primary fibroblast level, KFs exhibited increased TOMM20 and PINK1 expression, reduced Parkin expression, a decreased Parkin/PINK1 ratio, a reduced LC3B-II/LC3B-I ratio, and increased p62 expression compared with NFs (Figure 2H,I). However, because these markers were assessed under steady-state conditions without lysosomal inhibition, they cannot directly establish mitophagic flux. Taken together, these findings support the presence of defective PINK1/Parkin-dependent mitophagy in keloids, although the precise stage at which the mitophagy pathway is impaired remains to be determined.

### 3.3. Defective Mitophagy Promotes mtDNA Leakage Which Activates cGAS/STING-Mediated Innate Immunity

**Figure 3:**
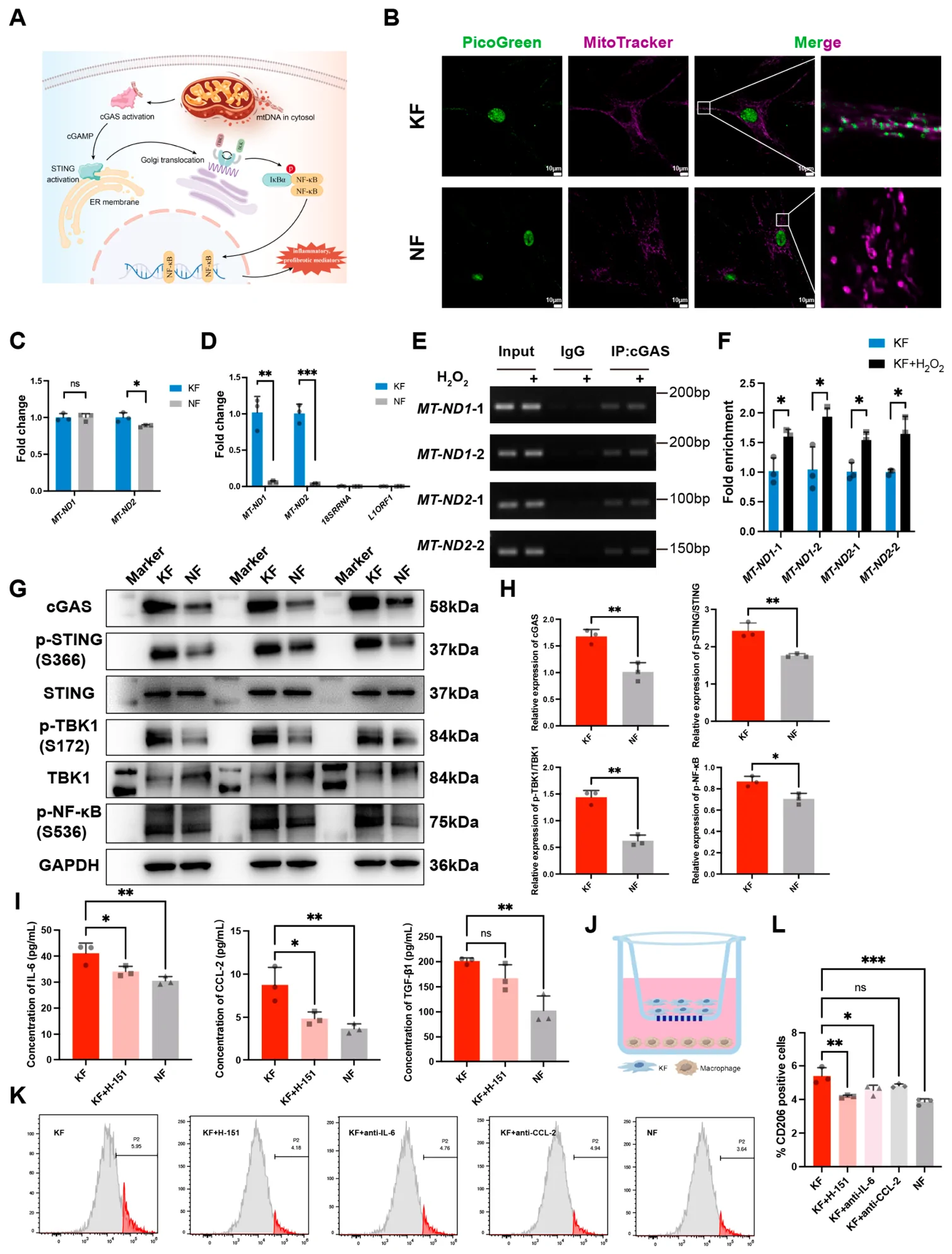
Defective mitophagy promotes mtDNA leakage, which activates cGAS/STING-mediated innate immunity. (**A**) Schematic illustration depicting the proposed mechanism whereby defective PINK1/Parkin-dependent mitophagy results in the accumulation of damaged mitochondria, cytosolic mtDNA leakage, and activation of the cGAS/STING/TBK1/NF-κB signaling pathway. (**B**) Representative confocal microscopy images of KFs and NFs stained with PicoGreen (dsDNA) and MitoTracker Deep Red (Mitochondria), showing mtDNA leakage. (**C**) Quantification of total mtDNA copy number using *MT-ND1* and *MT-ND2* qPCR assays. (**D**) Quantification of cytosolic mtDNA (*MT-ND1* and *MT-ND2*); in contrast, nuclear DNA markers (*18SRRNA* and *L1ORF1*) were undetectable, confirming the absence of nuclear DNA contamination. Total and cytosolic mtDNA datasets were independently normalized to their respective NF controls. Consequently, the fold-change values in (**C**) and (**D**) are not directly comparable and do not represent the cytosolic-to-total mtDNA ratio. The fractionation procedure depleted intact mitochondria, although a minor contribution from residual or disrupted mitochondria cannot be completely excluded. (**E**) Assessment of mtDNA bound to cGAS by PCR following immunoprecipitation in KFs with or without H_2_O_2_ treatment. Full uncropped gel is shown in Supplementary Figure S4B. (**F**) Quantitative analysis showing the enrichment of *MT-ND1* and *MT-ND2* in the cGAS immunoprecipitates relative to input. (**G**) Representative Western blot images showing cGAS/STING pathway-related proteins, including cGAS, STING, phospho-STING (p-STING), TBK1, phospho-TBK1 (p-TBK1), and phospho-NF-κB (p-NF-κB). Full uncropped blot is shown in Supplementary Figure S5. (**H**) Quantitative analysis of cGAS and p-NF-κB expression levels, as well as p-STING/STING and p-TBK1/TBK1 ratios shown in (**G**). (**I**) ELISA quantification of IL-6, CCL-2, and TGF-β1 levels in conditioned media from KFs, H-151-treated KFs, and NFs. (**J**) Schematic illustration of the Transwell co-culture system used to assess macrophage polarization. (**K**) Representative flow cytometric plots showing CD206-positive M2 macrophages after 48 h of co-culture with KFs, H-151-treated KFs, IL-6-neutralized KFs, CCL-2-neutralized KFs, or NFs. The red portion denotes CD206-positive M2 macrophages. (**L**) Quantitative analysis of M2 macrophage polarization rates. Data are presented as mean ± SD from *n* = 3 biological replicates per group. Statistical significance was determined using two-tailed Student’s *t*-test for comparisons between two groups and one-way ANOVA followed by Tukey’s multiple-comparisons test for comparisons among multiple groups. * *p* < 0.05, ** *p* < 0.01, *** *p* < 0.001.

Because damaged mitochondria are the major source of cytosolic mtDNA, we next investigated whether impaired mitophagy in KFs promotes cytosolic mtDNA leakage and subsequent activation of innate immune signaling. The proposed mechanism is illustrated in Figure 3A. Briefly, defective mitophagy leads to the persistence of damaged mitochondria, resulting in mtDNA escape into the cytosol, activation of the cGAS/STING signaling cascade, and production of downstream inflammatory and pro-fibrotic mediators. To determine whether mtDNA leakage occurs in KFs, cytosolic mtDNA was visualized by confocal microscopy using PicoGreen and MitoTracker staining. Compared with NFs, KFs exhibited markedly increased PicoGreen-positive DNA puncta that were spatially separated from the mitochondrial network, indicating enhanced mtDNA leakage into the cytosol (Figure 3B). To quantitatively assess mtDNA abundance, qPCR analysis was performed using primers targeting *MT-ND1* and *MT-ND2*. Total cellular mtDNA content showed no significant difference in *MT-ND1* copy number between NFs and KFs, whereas *MT-ND2* exhibited a modest increase in KFs (Figure 3C). In contrast, qPCR analysis of the mitochondria-depleted cytosolic fractions revealed significantly increased *MT-ND1* and *MT-ND2* abundance in KFs, whereas the nuclear DNA markers *L1ORF1* and *18SRRNA* were undetectable (Figure 3D). Because the total and cytosolic mtDNA datasets were independently normalized to their respective NF controls, the fold-change values shown in Figure 3C and 3D cannot be directly compared and do not indicate the proportion of total cellular mtDNA present in the cytosol. Together with the imaging findings, these results were consistent with increased cytosolic mtDNA accumulation in KFs; however, a minor contribution from residual or disrupted mitochondria could not be completely excluded. To directly determine whether defective PINK1/Parkin-dependent mitophagy contributes to mtDNA leakage, Parkin expression was silenced in NFs using si-*PRKN*. Confocal imaging revealed a pronounced increase in cytosolic mtDNA puncta following *PRKN* knockdown (Supplementary Figure S3A). Consistently, qPCR analysis showed significantly elevated levels of cytosolic *MT-ND1* and *MT-ND2* in si-*PRKN*-treated NFs compared with control NFs (Supplementary Figure S3B,C). These results provide evidence that disruption of PINK1/Parkin-dependent mitophagy can promote mtDNA leakage.

Cytosolic mtDNA is a well-recognized DAMP that activates innate immunity through binding to cGAS [14]. We therefore investigated whether mtDNA leakage triggers cGAS/STING signaling in KFs. CCK-8 analysis showed that 1 h exposure to 6 mmol/L H_2_O_2_ reduced KF metabolic viability to 41.8 ± 7.0% of the untreated control, indicating substantial acute metabolic impairment (Supplementary Figure S4A). cGAS-mtDNA immunoprecipitation assay was performed to assess the interaction between cGAS and mtDNA (Figure 3E,F, Supplementary Figure S4B,C). As shown in Figure 3E,F, both *MT-ND1* and *MT-ND2* were readily detected in cGAS immunoprecipitates. Importantly, induction of additional mtDNA leakage by H_2_O_2_ treatment further increased the enrichment of *MT-ND1* and *MT-ND2* within cGAS-bound complexes. These findings suggest that mtDNA can be recovered in association with cGAS under acute oxidative stress and support an association between cytosolic mtDNA accumulation and cGAS engagement; however, the substantial reduction in metabolic viability should be considered when interpreting the H_2_O_2_ challenge experiment. We next examined activation of the downstream cGAS/STING signaling pathway. Western blot analysis was used to evaluate the phosphorylation status of key signaling molecules (Figure 3G). Quantitative analysis showed significantly increased p-STING/STING and p-TBK1/TBK1 ratios in KFs compared with NFs. The relative abundance of p-NF-κB, normalized to GAPDH, was also increased in KFs (Figure 3G,H). Because cGAS/STING signaling is a central regulator of inflammatory and pro-fibrotic responses, we next quantified downstream cytokine secretion. ELISA analyses showed significantly increased production of IL-6, CCL-2, and TGF-β1 in KFs compared with NFs (Figure 3I). Notably, pharmacological inhibition of STING using H-151 significantly reduced the secretion of IL-6 and CCL-2, whereas TGF-β1 exhibited a downward trend without reaching statistical significance. These findings indicate that activation of the cGAS/STING pathway substantially contributes to the inflammatory secretory phenotype of KFs.

Given the emerging role of stromal–immune interactions in keloid progression, we further investigated whether activation of cGAS/STING signaling in KFs influences macrophage polarization. A Transwell co-culture system was established to mimic paracrine communication between fibroblasts and macrophages within the keloid microenvironment (Figure 3J). After 48 h of co-culture, flow cytometric analysis revealed that macrophages exposed to KFs exhibited a significantly higher proportion of CD206^+^ M2 macrophages than those exposed to NFs (Figure 3K,L). Importantly, inhibition of STING signaling by H-151 significantly attenuated KF-induced M2 polarization. Similarly, neutralization of IL-6 partially suppressed M2 polarization, whereas blockade of CCL-2 failed to produce a significant effect. These findings suggest that activation of the mtDNA-cGAS/STING axis in KFs promotes macrophage M2 polarization, at least in part through IL-6-dependent paracrine signaling. Taken together, these results identify defective PINK1/Parkin-dependent mitophagy as an upstream driver of mtDNA leakage in KFs. The released mtDNA activates cGAS/STING-mediated innate immune signaling, enhances inflammatory cytokine production, and promotes macrophage M2 polarization, thereby contributing to the establishment of a pro-fibrotic stromal–immune niche in keloids.

### 3.4. Transplanted Mitochondria Efficiently Engraft into KFs and Ameliorate Bioenergetic Deficits and Oxidative Stress

Building on the mitochondrial dysfunction identified above, we next asked whether exogenous mitochondria could be efficiently internalized by KFs and rescue their bioenergetic defects. Furthermore, we compared the responses of KFs to nMito and aMito administered at the same protein-equivalent dose. To establish the ADSC donor source, primary ADSCs were isolated from human lipoaspirate specimens by enzymatic digestion. Freshly isolated ADSCs at P0 and culture-expanded ADSCs at P3 displayed characteristic spindle-shaped fibroblastoid morphology (Supplementary Figure S6A,B). Multilineage differentiation assays confirmed their adipogenic and osteogenic differentiation capacities (Supplementary Figure S6C,D). Flow cytometric immunophenotyping demonstrated high surface expression of mesenchymal markers (CD105, CD90, CD73 ≥ 95%) and minimal expression of hematopoietic markers (CD45, CD14, CD34, CD19, HLA-DR < 2%) (Supplementary Figure S6E,F).

**Figure 4:**
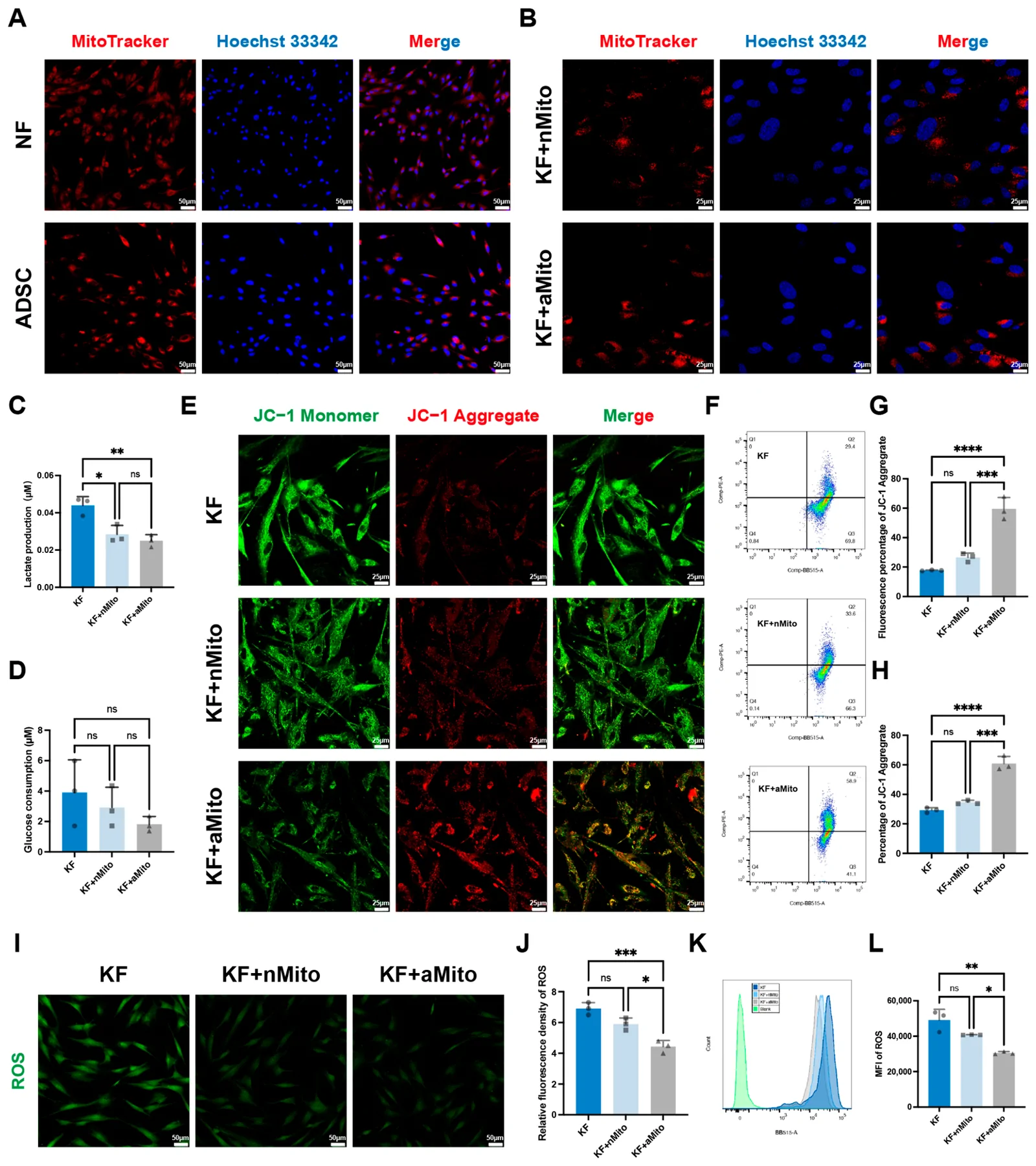
Transplanted mitochondria efficiently engraft into KFs and ameliorate bioenergetic deficits and oxidative stress. Normal skin fibroblast-derived mitochondria (nMito) and adipose-derived stem cell-derived mitochondria (aMito) were administered to KFs at a protein-equivalent dose of 25 μg mitochondrial protein per 1 × 10^5^ cells and incubated for 24 h. (**A**) Representative confocal microscopy images of donor NFs and ADSCs pre-labeled with MitoTracker Red prior to mitochondrial isolation. Mitochondria are shown in red and nuclei are counterstained with Hoechst 33342 (blue). (**B**) Representative confocal microscopy images showing internalization of MitoTracker Red-labeled donor mitochondria by KFs following co-culture. Exogenous mitochondria are shown in red. (**C**) Quantification of lactate production in KFs following transplantation of nMito or aMito. (**D**) Quantification of glucose consumption in conditioned medium from untreated KFs, nMito-treated KFs, and aMito-treated KFs. (**E**) Representative confocal microscopy images of JC-1 staining showing ΔΨm in untreated KFs, nMito-treated KFs, and aMito-treated KFs. (**F**) Representative flow cytometric histograms of JC-1-stained cells. (**G**) Quantification of ΔΨm measured by JC-1 fluorescent staining using confocal microscopy. (**H**) Quantification of ΔΨm measured by JC-1 fluorescent staining using flow cytometry. (**I**) Representative confocal microscopy images of DCFH-DA staining showing intracellular ROS levels in untreated KFs, nMito-treated KFs, and aMito-treated KFs. (**J**) Quantitative analysis of ROS fluorescence intensity shown in (**I**). (**K**) Representative flow cytometric histograms of ROS levels. (**L**) Quantitative analysis of ROS levels measured by flow cytometry shown in (**K**). Data are presented as mean ± SD from *n* = 3 biological replicates per group. Statistical significance was determined using one-way ANOVA followed by Tukey’s multiple-comparisons test. * *p* < 0.05, ** *p* < 0.01, *** *p* < 0.001, **** *p* < 0.0001.

To evaluate mitochondrial transplantation efficiency, donor NFs and ADSCs were pre-labeled with MitoTracker Red prior to mitochondrial isolation. Confocal microscopy confirmed robust mitochondrial labeling in both donor cell types (Figure 4A). Following isolation, labeled mitochondria were co-incubated with recipient KFs. Confocal imaging of co-cultured KFs revealed discrete intracellular red fluorescent puncta distributed within the recipient-cell cytoplasm (Figure 4B), supporting the uptake of donor-derived mitochondria from both donor sources. Having confirmed engraftment, we next evaluated the functional consequences of mitochondrial transplantation. First, we assessed whether mitochondrial transplantation could reverse metabolic reprogramming. Lactate production was quantified as a surrogate marker of glycolytic activity. Lactate production was significantly reduced in both nMito-treated (*p* < 0.05) and aMito-treated (*p* < 0.01) KFs compared with untreated KFs; aMito-treated cells exhibited a greater reduction than nMito-treated cells, though this difference did not reach statistical significance (Figure 4C). Measurement of glucose consumption revealed a downward trend from untreated KFs to nMito- and aMito-treated groups; however, these differences were not statistically significant (Figure 4D). This dissociation between lactate production and glucose consumption may reflect preferential restoration of mitochondrial oxidative metabolism, whereby pyruvate is increasingly directed toward mitochondrial oxidation rather than conversion to lactate.

We next evaluated ΔΨm as a direct indicator of mitochondrial functional integrity. JC-1 fluorescence was examined by confocal microscopy (Figure 4E) and flow cytometry (Figure 4F). Quantitative analysis of confocal images demonstrated that nMito transplantation induced only a non-significant increase in ΔΨm, while aMito transplantation produced a marked and statistically significant restoration of mitochondrial membrane polarization (Figure 4G). Quantitative flow cytometric analysis confirmed these findings, corroborating the confocal data through an independent measurement modality (Figure 4H). We then examined intracellular ROS levels to determine whether mitochondrial transplantation attenuates oxidative stress. DCFH-DA staining assessed by confocal microscopy showed markedly reduced ROS fluorescence in aMito-treated KFs, whereas the nMito group displayed only a modest and non-significant reduction (Figure 4I,J). Flow cytometric analysis confirmed these findings (Figure 4K,L). The consistent findings obtained from confocal microscopy and flow cytometry collectively indicate that mitochondrial transplantation effectively alleviates oxidative stress in KFs. Taken together, these findings reveal that exogenous mitochondria are efficiently internalized by KFs and functionally integrate to reverse glycolytic reprogramming, restore mitochondrial membrane polarization, and suppress oxidative stress.

### 3.5. Mitochondrial Transplantation Enhances PINK1/Parkin-Dependent Mitophagy and Limits Cytosolic mtDNA Accumulation

**Figure 5:**
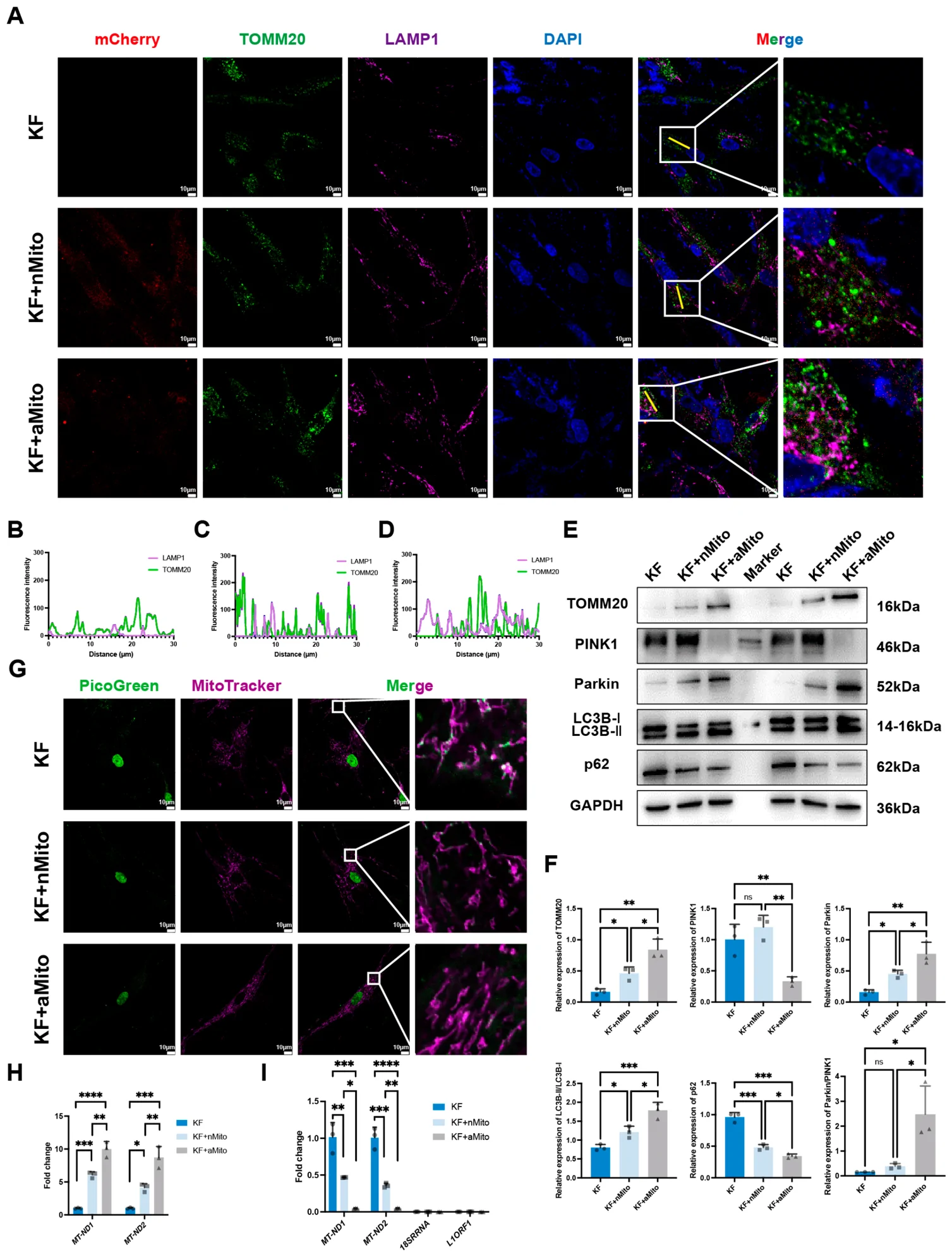
Mitochondrial transplantation enhances PINK1/Parkin-dependent mitophagy and limits cytosolic mtDNA accumulation. (**A**) Representative confocal microscopy images showing mitochondria-lysosome colocalization in untreated KFs, nMito-treated KFs, and aMito-treated KFs. Donor-derived mitochondria were labeled with mitochondria-targeted mCherry (red), the total mitochondrial population was stained for TOMM20 (green), lysosomes were stained for LAMP1 (purple), and nuclei were counterstained with DAPI (blue). The yellow lines indicate the regions used for the quantitative analyses shown in (**B**–**D**). (**B**–**D**) Quantitative analyses of mitochondria-lysosome colocalization in KFs (**B**), nMito-treated KFs (**C**), and aMito-treated KFs (**D**). These panels show spatial fluorescence overlap along the selected lines and should not be interpreted as whole-cell colocalization coefficients. (**E**) Representative Western blot images showing expression of TOMM20, PINK1, Parkin, LC3B, and p62 in untreated KFs, nMito-treated KFs, and aMito-treated KFs. Full uncropped blot is shown in Supplementary Figure S7A. (**F**) Quantitative analysis of protein expression levels shown in (**E**). (**G**) Representative confocal images of cells stained with PicoGreen for double-stranded DNA (green) and MitoTracker Deep Red for mitochondria (magenta). Enlarged regions show PicoGreen-positive signals spatially separated from the mitochondrial network. (**H**) Quantification of total mtDNA copy number using *MT-ND1* and *MT-ND2* qPCR assays. (**I**) Quantification of cytosolic mtDNA levels determined by qPCR; nuclear DNA markers (*18SRRNA* and *L1ORF1*) were concurrently assessed and were undetectable. Data are presented as mean ± SD from *n* = 3 biological replicates per group. Statistical significance was determined using one-way ANOVA followed by Tukey’s multiple-comparisons test. * *p* < 0.05, ** *p* < 0.01, *** *p* < 0.001, **** *p* < 0.0001.

Our preceding findings established that KFs harbor a profound blockade of PINK1/Parkin-dependent mitophagy, which leads to the accumulation of damaged mitochondria and subsequent cytosolic leakage of mtDNA. Intriguingly, emerging evidence indicates that exogenous mitochondrial transplantation can not only replenish functional mitochondrial pools and normalize recipient cell bioenergetics but also actively stimulate endogenous mitophagy pathways [19]. Inspired by these insights, we next sought to investigate whether mitochondrial transplantation could facilitate the clearance of damaged organelles by rescuing the defective PINK1/Parkin-dependent mitophagy in KFs. To distinguish transplanted mitochondria from endogenous mitochondria of KFs, donor NFs and ADSCs were transduced with a mitochondria-targeted mCherry lentiviral construct prior to mitochondrial isolation. Freshly isolated mCherry-labeled mitochondria were subsequently co-cultured with KFs. To assess mitochondrial trafficking toward lysosomes, confocal immunofluorescence was performed using TOMM20 to visualize the total mitochondrial population and LAMP1 to label lysosomes, while transplanted donor-derived mitochondria were identified by mitochondria-targeted mCherry fluorescence (Figure 5A). Mitochondrial transplantation increased mitochondria-lysosome colocalization compared with untreated KFs (Figure 5B–D), indicating enhanced delivery or association of mitochondria with lysosomal compartments. Western blot analysis further showed reduced PINK1 expression, increased Parkin expression and Parkin/PINK1 ratio, an increased LC3B-II/LC3B-I ratio and reduced p62 expression following mitochondrial transplantation (Figure 5E,F). Notably, aMito consistently produced greater changes in these mitophagy-associated markers than nMito.

Because defective mitophagy has been linked to mtDNA leakage in KFs, we next examined whether enhanced mitophagy induced by mitochondrial transplantation could reduce cytosolic mtDNA accumulation. Confocal imaging showed a marked reduction in cytosolic mtDNA puncta following mitochondrial transplantation (Figure 5G). qPCR analysis revealed progressive increases in total mtDNA copy number, as reflected by elevated *MT-ND1* and *MT-ND2* levels after transplantation (Figure 5H). In contrast, cytosolic mtDNA abundance was significantly reduced, with both *MT-ND1* and *MT-ND2* levels being highest in untreated KFs, intermediate in nMito-treated KFs, and lowest in aMito-treated KFs (Figure 5I). To provide an mtDNA-independent biochemical assessment, we measured citrate synthase activity in whole-cell lysates. Citrate synthase activity was significantly increased following both nMito and aMito transplantation, with aMito producing a greater increase than nMito (Supplementary Figure S7B). These findings suggest that mitochondrial transplantation replenishes mitochondrial content while limiting pathological mtDNA leakage into the cytosol. Collectively, these findings indicate that mitochondrial transplantation restores mitochondrial quality control through activation of PINK1/Parkin-dependent mitophagy, thereby limiting cytosolic mtDNA accumulation in KFs.

### 3.6. Mitochondrial Transplantation Suppresses cGAS/STING Signaling and Inflammatory Remodeling

**Figure 6:**
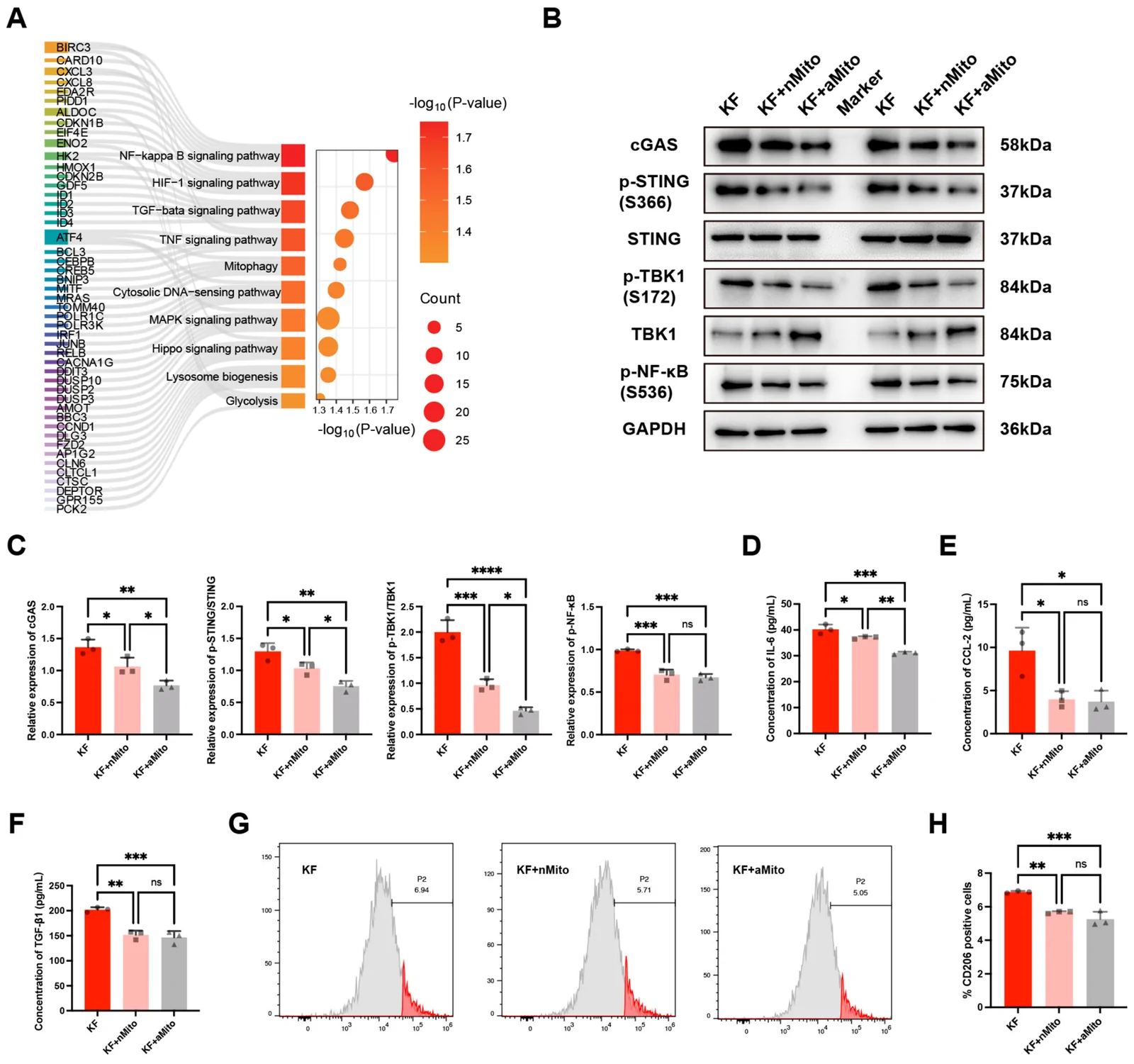
Mitochondrial transplantation suppresses cGAS/STING signaling and inflammatory remodeling. (**A**) Sankey and enrichment-dot plot derived from exploratory bulk RNA sequencing of three donor-matched pairs of untreated and aMito-treated KFs (*n* = 3 independent KF donors per condition). The nMito condition was not included in this RNA-sequencing experiment. Connections link differentially expressed genes to selected enriched pathways; dot size represents gene count and dot color represents −log_10_. (**B**) Representative Western blot images showing cGAS/STING pathway-related proteins, including cGAS, STING, p-STING, TBK1, p-TBK1, and p-NF-κB, in untreated KFs, nMito-treated KFs, and aMito-treated KFs. Full uncropped blot is shown in Supplementary Figure S8. (**C**) Quantitative analysis of cGAS and p-NF-κB expression levels, as well as p-STING/STING and p-TBK1/TBK1 ratios shown in (**B**). (**D**–**F**) ELISA quantification of IL-6 (**D**), CCL-2 (**E**), and TGF-β1 (**F**) levels in conditioned media from untreated KFs, nMito-treated KFs, and aMito-treated KFs. (**G**) Representative flow cytometric plots showing CD206-positive macrophages following co-culture with untreated KFs, nMito-treated KFs, or aMito-treated KFs. The red portion denotes CD206-positive M2 macrophages. (**H**) Quantitative analysis of CD206-positive macrophage percentages shown in (**G**). Data are presented as mean ± SD from *n* = 3 biological replicates per group. Statistical significance was determined using one-way ANOVA followed by Tukey’s multiple-comparisons test. * *p* < 0.05, ** *p* < 0.01, *** *p* < 0.001, **** *p* < 0.0001.

To further investigate the molecular changes associated with aMito treatment, bulk RNA sequencing was performed. The nMito condition was not included in this RNA-seq experiment. Pathway enrichment analysis revealed exploratory associations with glycolysis-, mitophagy-, and lysosome biogenesis-related pathways (Figure 6A). Notably, pathways related to cytosolic DNA sensing and NF-κB signaling were also identified in this exploratory analysis. Given that mitochondrial transplantation reduced cytosolic mtDNA accumulation in KFs, these exploratory transcriptomic associations raised the possibility that suppression of mtDNA leakage may attenuate cGAS/STING-mediated innate immune signaling. To test this hypothesis, we next examined the activation status of the cGAS/STING pathway following mitochondrial transplantation. Western blot analysis demonstrated that mitochondrial transplantation suppressed activation of the cGAS/STING signaling cascade in KFs (Figure 6B). Quantitative analysis revealed a stepwise reduction in cGAS expression following nMito and aMito transplantation, with significantly lower levels observed in both treatment groups compared with untreated KFs and a further decrease in the aMito group relative to the nMito group (Figure 6C). The phosphorylation levels of STING and TBK1 were reduced after mitochondrial transplantation, indicating attenuation of downstream signal transduction. Notably, aMito treatment produced significantly greater suppression of p-STING/STING and p-TBK1/TBK1 compared with nMito treatment. The relative abundance of p-NF-κB was lower in both mitochondria-treated groups than in untreated KFs, whereas no significant difference was observed between the nMito and aMito groups. These data indicate that mitochondrial transplantation may reduce mtDNA-cGAS/STING pathway activation.

Because activation of the cGAS/STING pathway promotes the production of inflammatory and pro-fibrotic mediators, we next evaluated whether suppression of this pathway translates into reduced inflammatory remodeling. ELISA analysis revealed that mitochondrial transplantation significantly decreased the secretion of IL-6, CCL-2, and TGF-β1 by KFs (Figure 6D–F). IL-6 levels were reduced from untreated KFs to nMito- and aMito-treated groups, with aMito producing a significantly greater reduction than nMito. Likewise, secretion of CCL-2 and TGF-β1 was significantly attenuated following both mitochondrial transplantation treatments. Although the aMito group consistently exhibited lower cytokine levels than the nMito group, these differences did not reach statistical significance for CCL-2 or TGF-β1. These findings indicate that suppression of cGAS/STING signaling is accompanied by attenuation of the inflammatory and pro-fibrotic secretory phenotype of KFs. Given our previous observation that fibroblast-derived mediators promote macrophage M2 polarization, we further examined whether mitochondrial transplantation modulates stromal–immune communication within the keloid microenvironment. Macrophages were exposed to untreated or mitochondria-treated KFs via the same Transwell system described above (Figure 6G). Flow cytometric analysis demonstrated that conditioned signaling from mitochondria-treated KFs induced significantly fewer CD206^+^ M2 macrophages than untreated KFs (Figure 6H). Both nMito and aMito transplantation significantly reduced KF-induced M2 polarization, whereas no significant difference was detected between the two treatment groups. Taken together, these results demonstrate that mitochondrial transplantation not only suppresses cGAS/STING-mediated innate immune activation within KFs but also attenuates downstream inflammatory remodeling and pro-fibrotic stromal–immune interactions.

### 3.7. Mitochondrial Transplantation Inhibits Proliferation, Migration, Invasion, and Collagen Synthesis In Vitro

**Figure 7:**
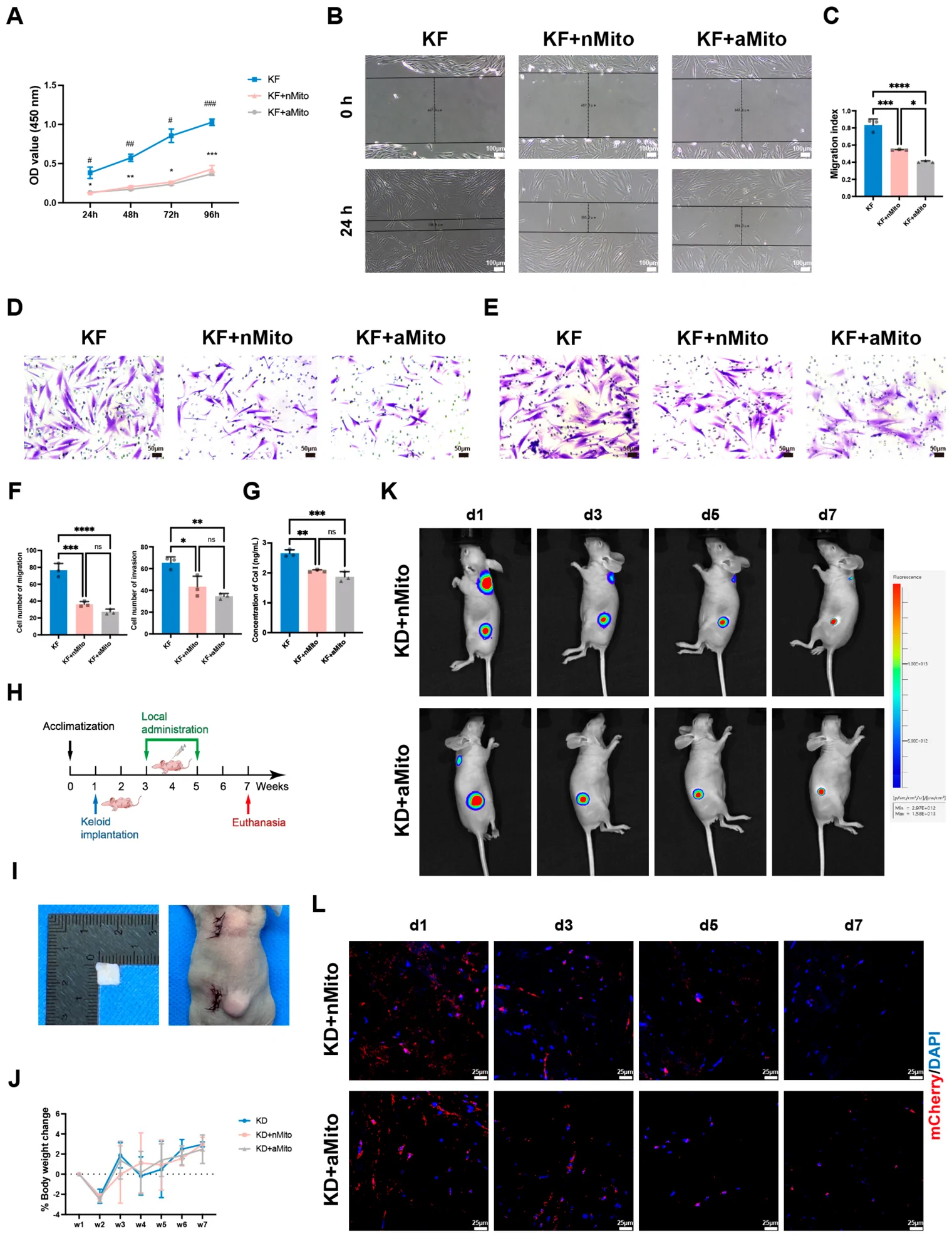
Mitochondrial transplantation suppresses fibroblast activation in vitro and demonstrates in vivo lesion retention. (**A**) CCK-8 assays showing proliferation of untreated KFs, nMito-treated KFs, and aMito-treated KFs at 24, 48, 72, and 96 h. (**B**) Representative scratch wound-healing images at 0 and 24 h. (**C**) Quantification of wound closure rates from (**B**). (**D**) Representative Transwell migration images. (**E**) Representative Matrigel invasion images. (**F**) Quantification of migrated and invaded cells from (**D**,**E**). (**G**) ELISA quantification of type I collagen (Col I) secretion in conditioned media from untreated KFs, nMito-treated KFs, and aMito-treated KFs. (**H**) Schematic timeline of the keloid (KD) xenograft experiment and intrafocal mitochondria administration. Female BALB/c nude mice received subcutaneous human keloid implants and were subsequently treated by multipoint intralesional injection of vehicle, nMito, or aMito twice weekly for 2 weeks, followed by a 2-week observation period. (**I**) Representative images of standardized keloid pieces before implantation and immediately after subcutaneous implantation in nude mice. (**J**) Percentage change in body weight during the treatment period. (**K**) In vivo fluorescence imaging of xenografts after intrafocal injection of mCherry-labeled nMito or aMito at days 1, 3, 5, and 7 post-injection. (**L**) Representative confocal images showing retained exogenous mitochondria (red) within xenograft tissues; nuclei were counterstained with DAPI (blue). For the in vitro quantitative analyses (**A**,**C**,**F**,**G**), *n* = 3 biological replicates per group. For body-weight analysis (**J**), *n* = 6 mice per group. Mitochondrial retention analyses (**K**,**L**) were performed in an additional cohort of *n* = 2 mice per time point for qualitative assessment. Statistical significance was determined using one-way or two-way ANOVA followed by Tukey’s multiple-comparisons test. *^, #^ *p* < 0.05, **^, ##^ *p* < 0.01, ***^, ###^ *p* < 0.001, **** *p* < 0.0001.

Having elucidated the underlying molecular mechanisms of mitochondrial transplantation in KFs, we next investigated whether these molecular changes translate into attenuation of the activated fibroblast phenotype. CCK-8 assays demonstrated a sustained suppression of KF growth following mitochondrial transplantation (Figure 7A). At 24, 48, 72, and 96 h, cell viability remained significantly lower in both the nMito- and aMito-treated groups than in untreated KFs, whereas no significant difference was observed between the two mitochondrial donor groups. Scratch wound-healing assays and subsequent quantitative analysis confirmed that untreated KFs closed the wound area more rapidly than mitochondria-treated KFs, with the aMito group exhibiting a stronger inhibitory effect on wound closure than the nMito group (Figure 7B,C). We further evaluated directed migration and invasive behavior using Transwell assays. Representative images are shown in Figure 7D,E. Quantification revealed that both nMito and aMito reduced chemotactic migration relative to untreated KFs, whereas the difference between the two donor groups was not significant (Figure 7F). A similar pattern was observed for Matrigel invasion, with significant reductions after nMito and aMito treatment compared with untreated KFs, but no significant difference between nMito and aMito (Figure 7F). Because excessive matrix production is a defining feature of KFs, we next quantified type I collagen secretion by ELISA. Conditioned media from mitochondria-treated KFs contained less Col I than media from untreated KFs, whereas the difference between nMito and aMito was not significant (Figure 7G). Collectively, both mitochondrial treatments attenuated multiple pathogenic KF phenotypes. Differences between aMito and nMito were observed only for selected outcomes under the protein-normalized dosing conditions used in this study.

### 3.8. Mitochondrial Transplantation Mitigates Keloid Fibrosis and Innate Immune Activation In Vivo

To determine whether the in vitro effects of mitochondrial transplantation could be reproduced in vivo, we established a human keloid xenograft model in BALB/c nude mice and administered intralesional vehicle, nMito, or aMito according to the schedule shown in Figure 7H. Representative images of the implanted keloid pieces and the immediate postoperative appearance are presented in Figure 7I. Throughout the treatment period, body weight remained stable in all groups, and the percentage change in body weight did not differ significantly among vehicle-, nMito-, and aMito-treated mice (Figure 7J), indicating that repeated intralesional mitochondria administration was well tolerated. We next examined the persistence of transplanted mitochondria within the xenografts. In vivo fluorescence imaging of lesions injected with mCherry-labeled mitochondria demonstrated detectable signals at days 1, 3, 5, and 7 after injection in both the nMito and aMito groups, with progressive signal attenuation over time (Figure 7K). Confocal imaging of cryosections confirmed the presence of red fluorescent exogenous mitochondria within the keloid tissue after injection (Figure 7L). These observations indicate that transplanted mitochondria can be retained within the keloid lesion for at least one week following intrafocal administration.

**Figure 8:**
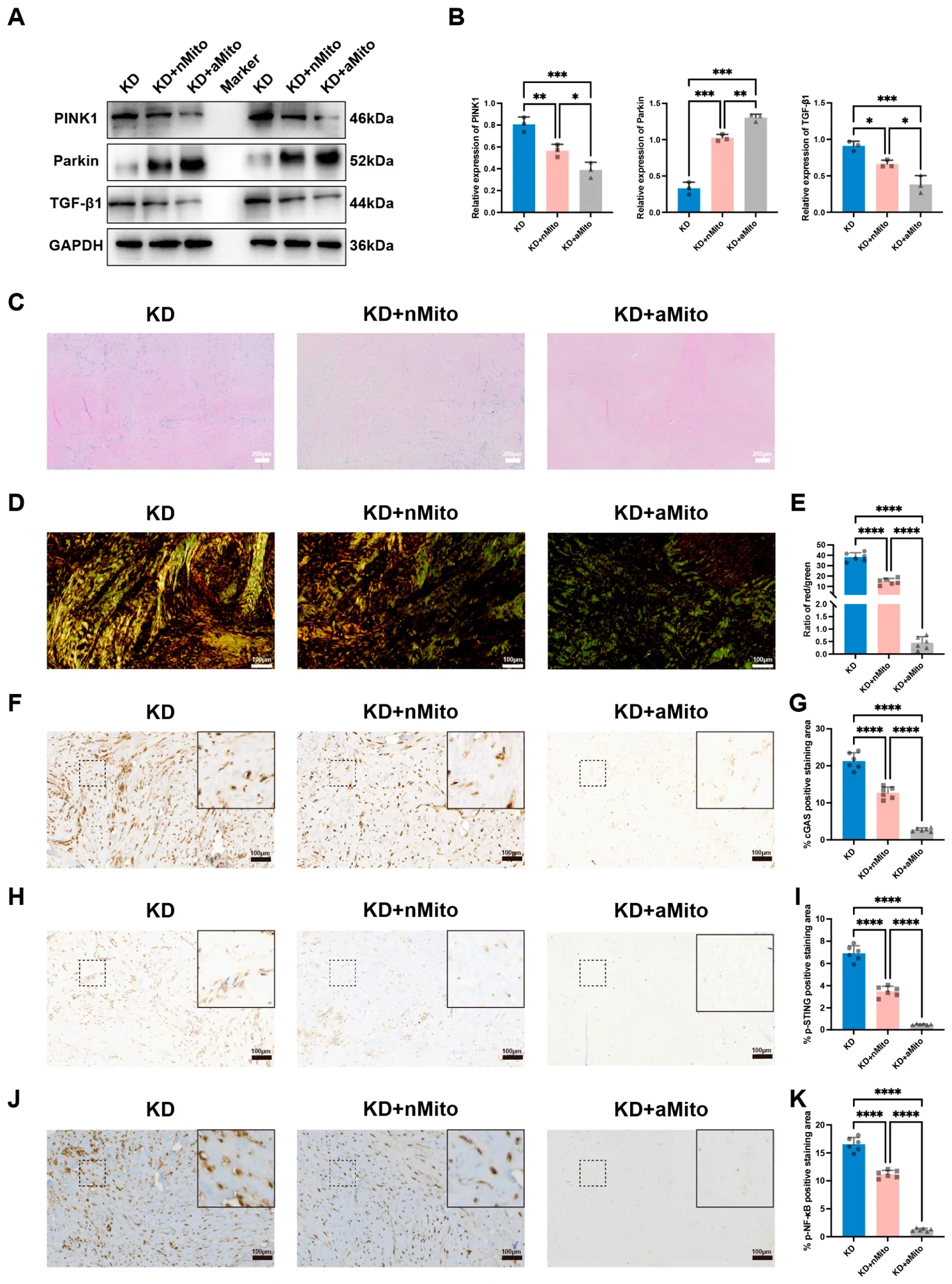
Mitochondrial transplantation attenuates fibrosis and suppresses innate immune activation in keloid xenografts. Female BALB/c nude mice bearing subcutaneous human keloid xenografts received multipoint intralesional injections of vehicle, nMito, or aMito twice weekly for 2 weeks. Vehicle-treated lesions received 50 μL PBS, whereas mitochondria-treated lesions received 40 μg mitochondria in 50 μL PBS per injection. KD denotes vehicle-treated keloid xenografts; KD+nMito and KD+aMito denote nMito- and aMito-treated xenografts, respectively. (**A**) Representative Western blot images showing PINK1, Parkin, and TGF-β1 expression in vehicle-, nMito-, and aMito-treated xenografts. Full uncropped blot is shown in Supplementary Figure S9. (**B**) Quantitative analysis of protein expression levels shown in (**A**). (**C**) Representative H&E staining of xenograft tissues. (**D**) Representative Sirius Red staining showing fibrillar collagen deposition and organization. (**E**) Quantification of the red/green birefringent fibre area ratio, calculated as the red-orange/yellow birefringent collagen area divided by the green birefringent collagen area. (**F**) Representative immunohistochemical staining for cGAS. (**G**) Quantitative analysis of cGAS staining. (**H**) Representative immunohistochemical staining for p-STING. (**I**) Quantitative analysis of p-STING staining. (**J**) Representative immunohistochemical staining for p-NF-κB. (**K**) Quantitative analysis of p-NF-κB staining. Data are presented as mean ± SD (*n* = 3 mice per group for Western blot analysis and *n* = 6 mice per group for histological and immunohistochemical staining). Statistical significance was determined using one-way ANOVA followed by Tukey’s multiple-comparisons test. * *p* < 0.05, ** *p* < 0.01, *** *p* < 0.001, **** *p* < 0.0001.

To provide a direct implant-level endpoint, initial and terminal xenograft weights were compared (Supplementary Figure S10). Initial implant weights were comparable among the vehicle, nMito, and aMito groups (89.1 ± 2.1, 88.2 ± 2.2, and 88.9 ± 1.4 mg, respectively; *p* = 0.687). At harvest, implant weights were 48.6 ± 4.6 mg in the vehicle group, 44.7 ± 7.9 mg in the nMito group, and 40.1 ± 4.4 mg in the aMito group. The absolute reduction in implant weight was greater in the aMito group than in the vehicle group, whereas the differences between nMito and vehicle and between aMito and nMito were not statistically significant. Having verified in vivo retention and documented implant-level weight changes, we next assessed molecular markers associated with PINK1/Parkin-dependent mitophagy and fibrosis. Western blot analysis of harvested xenografts showed that mitochondrial transplantation reduced PINK1 accumulation while increasing Parkin expression and suppressing TGF-β1 compared with vehicle-treated xenografts (Figure 8A,B). Histological evaluation further suggested attenuation of the fibrotic phenotype after mitochondrial transplantation. H&E staining showed a less compact and more organized dermal architecture in mitochondria-treated xenografts than in vehicle controls (Figure 8C). Sirius Red staining showed alterations in collagen fibre birefringence and organization after both nMito and aMito treatment (Figure 8D). Quantitative analysis showed a reduction in the red-orange/yellow-to-green birefringent collagen fibre area ratio in both treatment groups, with a greater reduction in the aMito group (Figure 8E). Because our in vitro studies identified the mtDNA-cGAS/STING pathway as a key mediator of innate immune activation, we next examined pathway activity in xenograft tissues. Immunohistochemistry performed in the same staining batch and evaluated in matched viable xenograft regions showed lower cGAS, p-STING, and p-NF-κB immunoreactivity in both mitochondria-treated groups than in vehicle controls (Figure 8F–K). Higher-magnification images confirmed preserved hematoxylin counterstaining, although the aMito-treated xenografts exhibited lower cellularity; therefore, these findings were interpreted as supportive histological evidence of reduced pathway-related immunoreactivity rather than definitive protein-level confirmation of pathway suppression. Taken together, these in vivo data indicate that transplanted mitochondria persist within keloid lesions, are well tolerated after repeated intralesional administration, restore the PINK1/Parkin mitophagy axis, attenuate fibrotic remodeling, and suppress cGAS/STING-mediated innate immune activation.

## 4. Discussion

Keloids are increasingly recognized as chronic inflammatory fibrotic disorders in which mitochondrial dysfunction and immune activation are closely intertwined, yet the mechanistic link between impaired mitochondrial quality control and chronic inflammation has remained unclear [1,5]. In the present study, we demonstrate that defective PINK1/Parkin-dependent mitophagy drives mtDNA-cGAS/STING-mediated innate immune activation in keloids and that mitochondrial transplantation restores mitochondrial homeostasis to suppress this pathogenic signaling axis. Furthermore, we identify ADSC-derived mitochondria as a more effective donor source than normal skin fibroblast-derived mitochondria, providing a potential organelle-based therapeutic strategy for keloid fibrosis.

Maintenance of mitochondrial quality is essential for preserving cellular homeostasis [8]. Mitochondrial quality control prevents the accumulation of dysfunctional mitochondria that would otherwise amplify oxidative stress, inflammatory signaling, and tissue fibrosis [33]. Among the multiple quality-control mechanisms, PINK1/Parkin-dependent mitophagy represents the best-characterized pathway responsible for the selective elimination of damaged mitochondria [12]. Upon loss of mitochondrial membrane potential, PINK1 is stabilized on the outer mitochondrial membrane, where it recruits and activates Parkin to ubiquitinate mitochondrial substrates and initiate mitophagy. Efficient coordination between PINK1 accumulation and Parkin recruitment is therefore essential for effective mitophagy. Interestingly, we found that although PINK1 expression was elevated in keloids, Parkin expression was significantly reduced, indicating that mitophagic initiation and execution become uncoupled. This imbalance likely results in defective clearance of dysfunctional mitochondria and progressive accumulation of mitochondrial damage. Notably, tissue immunohistochemistry showed no significant difference in LC3B-II staining, whereas the LC3B-II/LC3B-I ratio was reduced in isolated KFs. This apparent discrepancy likely reflects the non-equivalence of a semiquantitative staining signal obtained from heterogeneous tissue and a fibroblast-specific ratio determined by the abundance of both LC3B forms, with tissue heterogeneity and in vitro culture conditions potentially contributing to the different patterns. Consistent with a previous study demonstrating impaired mitochondrial function and abnormal mitochondrial morphology in KFs, we observed extensive mitochondrial swelling and cristae disruption [11]. Our additional measurements showed mitochondrial membrane depolarization and increased intracellular oxidation-sensitive fluorescence in KFs. Recent studies have highlighted the context-dependent role of mitophagy in inflammation [16,34]. Notably, NF-κB, a master regulator of inflammatory responses, can induce p62-dependent mitophagy as a negative-feedback mechanism to restrain excessive inflammation [35]. However, the concurrent reduction in Parkin expression observed in KFs likely uncouples this compensatory response, rendering p62 accumulation insufficient to support mitophagy. Conversely, impaired mitophagy facilitates the persistence of damaged mitochondria and amplifies inflammatory signaling [36]. Therefore, our findings support the concept that mitophagy dysfunction represents a critical upstream event mediating the transition from mitochondrial damage to chronic inflammation in keloids.

A principal finding of this study was the coexistence of cytosolic mtDNA accumulation and cGAS/STING pathway activation in KFs. *PRKN* knockdown further increased cytosolic mtDNA, while H-151 attenuated selected inflammatory responses, supporting the involvement of Parkin- and STING-associated processes. Cytosolic mtDNA functions as a DAMP that is recognized by cGAS, triggering STING activation and downstream TBK1 and NF-κB signaling. Aberrant activation of this pathway has been implicated in multiple fibrotic disorders [37]. Consistent with these observations, we detected increased cytosolic mtDNA accumulation, enhanced mtDNA-cGAS interaction, and activation of STING, TBK1, and NF-κB signaling in KFs. Importantly, genetic suppression of Parkin was able to induce mtDNA leakage, supporting a causal relationship between PINK1/Parkin-dependent mitophagy deficiency and cGAS/STING activation. Beyond its role as a downstream effector, NF-κB occupies a central position in coordinating inflammatory and fibrotic responses [38]. Activation of NF-κB promotes the transcription of multiple cytokines and chemokines that contribute to immune cell recruitment, fibroblast activation, and ECM remodeling. Therefore, activation of the mtDNA-cGAS/STING axis may represent a key mechanism sustaining chronic inflammation and fibrosis in keloids.

Another implication of this study relates to stromal–immune cell crosstalk during keloid progression. In keloid research, macrophages have traditionally been regarded as primary drivers of inflammation and fibrotic remodeling, with comparatively limited attention paid to the upstream stromal signals that shape immune-cell behavior [39]. In contrast, our findings place fibroblasts at the upstream position of this regulatory network, suggesting that mitochondrial dysfunction within stromal cells actively shapes immune-cell behavior. Stromal cells regulate immune-cell recruitment and activation through cytokine and chemokine networks [40]. In fibrotic tissues, stromal cells can orchestrate immune responses and establish self-perpetuating inflammatory circuits [41]. Skin fibroblasts, in particular, are highly dynamic cells capable of directing immune-cell trafficking and polarization during wound repair and fibrosis [42]. Our co-culture experiments demonstrated that activation of the mtDNA-cGAS/STING pathway in KFs promoted macrophage M2 polarization. Importantly, pharmacological inhibition of STING significantly attenuated this effect, indicating that fibroblast-intrinsic innate immune activation can influence macrophage phenotype. Among the cytokines examined, IL-6 appeared to play an important role. Neutralization of IL-6 partially suppressed M2 polarization, whereas CCL-2 blockade showed limited effects. These observations are consistent with recent reports demonstrating that macrophage STING signaling promotes fibrosis through activation of an IL-6-STAT3 axis [43]. Beyond its classical inflammatory functions, IL-6 has been increasingly recognized as a potent pro-fibrotic mediator capable of enhancing fibroblast activation, ECM production, and immune-cell polarization. Together, these findings expand the current understanding of keloid pathogenesis from a fibroblast-centered perspective toward a bidirectional stromal–immune interaction model driven by mitochondrial innate immune signaling.

Under the protein-normalized dosing conditions used in this study, aMito produced larger changes than nMito in selected outcomes, whereas several other endpoints showed no significant difference between the two preparations. These findings indicate donor-source-associated differences in the observed responses but do not establish the intrinsic superiority of aMito. Because protein normalization does not equalize mitochondrial number, purity, integrity, or respiratory competence, direct preparation-level characterization is required before differences can be attributed to donor-cell origin. Mitochondria are recognized as heterogeneous organelles whose metabolic characteristics depend on tissue origin [44]. Differences in mitochondrial respiratory capacity, stress resistance, mtDNA integrity, and redox regulation have been reported among distinct donor cell types [45]. ADSCs reside in a physiologically dynamic microenvironment and exhibit high metabolic adaptability, robust mitochondrial biogenesis, and enhanced resistance to oxidative stress [46]. These properties may provide a more functional mitochondrial pool capable of restoring mitochondrial homeostasis in recipient KFs. In addition, ADSCs possess well-established immunomodulatory properties [47]. Although isolated mitochondria lack the full secretory repertoire of parent cells, mitochondria derived from ADSCs may retain distinct metabolic and signaling characteristics that facilitate suppression of inflammatory responses after transplantation [48]. Importantly, ADSCs are readily accessible through minimally invasive procedures and provide abundant mitochondrial yields, making them particularly attractive for future translational applications [49]. The superior performance of aMito observed in the present study supports further preclinical optimization and evaluation of ADSC-derived mitochondria as a candidate organelle-based therapy for keloids and other fibrotic skin disorders. Moreover, previous studies demonstrated that frozen or otherwise inactivated mitochondria, mitochondrial fractions, and exogenous ATP/ADP failed to reproduce the protective effects of freshly isolated, respiration-competent mitochondria [50,51,52,53]. These findings support the importance of mitochondrial structural and functional integrity. Nevertheless, because an inactivated-mitochondria control was not included in the present study, a contribution from soluble constituents of the preparations cannot be formally excluded.

Potential limitations of the present study warrant discussion. First, although we demonstrated that KF-derived signals regulate macrophage polarization, the reciprocal effects of polarized macrophages on fibroblast activation were not investigated. The stromal–immune interaction network in keloids is likely bidirectional and considerably more complex than the current experimental model captures. Second, although activation of the mtDNA-cGAS/STING pathway was demonstrated, the downstream transcriptional programs responsible for fibrosis promotion remain incompletely defined. In particular, the involvement of IL-6-STAT3 signaling and other downstream effectors warrants further mechanistic investigation. Third, the long-term fate, persistence, and functional integration of transplanted mitochondria remain poorly understood. Whether exogenous mitochondria establish durable bioenergetic reprogramming or require repeated administration for sustained efficacy requires further study. Fourth, only female mice were included, and the human donor groups were not sex-matched, precluding evaluation of potential sex-dependent effects. Moreover, mitochondria derived from both NFs and ADSCs were allogeneic to recipient KFs, while donor–recipient mtDNA haplotype mismatch and the potential immunogenicity of mitochondrial N-formyl peptides were not assessed. The immunodeficient nude-mouse model also precluded adequate evaluation of adaptive allogeneic immune responses. Future studies should investigate the reciprocal interactions between mitochondria-treated fibroblasts and immune-cell subsets, further dissect downstream signaling networks, and employ single-cell and spatial transcriptomic approaches to resolve cell-type-specific mitochondrial responses. In addition, optimization of mitochondrial delivery strategies, donor selection, and long-term safety evaluation will be essential for clinical translation.

## 5. Conclusions

KFs exhibited defective PINK1/Parkin-dependent mitophagy, accompanied by cytosolic mtDNA accumulation and activation of the cGAS/STING/TBK1/NF-κB pathway, collectively supporting an association between mitochondrial homeostasis disruption and keloid inflammation and fibrosis. Mitochondrial transplantation was associated with improved mitochondrial function, changes in mitophagy-related markers, reduced mtDNA-cGAS/STING signaling, and attenuated fibroblast activation and inflammatory remodeling. Selected outcomes differed between ADSC- and NF-derived mitochondria under protein-normalized dosing conditions, although their relative intrinsic efficacy remains to be established. These findings provide evidence of an association between mitochondrial quality control and innate immune activation in keloids and support mitochondrial transplantation as a promising organelle-based immunometabolic strategy for fibrotic skin disease.

## Data Availability

The publicly available dataset GSE145725 was obtained from the NCBI Gene Expression Omnibus (GEO) database and was used for secondary bioinformatic analysis. The newly generated RNA sequencing dataset supporting the findings of this study has been deposited in the GEO database under accession number GSE343389. All other datasets generated or analyzed during this study are included in the main text or the Supplementary Materials.

## Supplementary Materials

The following supporting information can be downloaded at: https://www.mdpi.com/article/10.3390/antiox15091120/s1, Supplementary Table S1: Demographic and clinical characteristics of the human tissue donors; Supplementary Table S2: Primer sequences; Supplementary Figure S1: Quantitative analyses of mitochondrial bioenergetics, membrane potential, oxidative stress, and bioinformatic validation in KFs. (A) Quantification of Seahorse mitochondrial stress test parameters, including basal respiration, maximal respiration, ATP production, and spare respiratory capacity. (B,C) Quantification of ΔΨm measured by JC-1 fluorescent staining using confocal microscopy (B) and flow cytometry (C). (D,E) Quantification of ROS levels measured by confocal microscopy (D) and flow cytometry (E), respectively. (F) Box plot showing relative PINK1 transcript expression in KFs versus NFs derived from the GEO dataset GSE145725. Data are presented as mean ± SD from *n* = 3 biological replicates per group. Statistical significance was determined using two-tailed Student’s *t*-test. * *p* < 0.05, ** *p* < 0.01, *** *p* < 0.001, **** *p* < 0.0001; Supplementary Figure S2: Full uncropped blot for Figure 2H; Supplementary Figure S3: Defective PINK1/Parkin-dependent mitophagy promotes mtDNA leakage. (A) Representative confocal microscopy images of control NFs and *PRKN*-silenced NFs (NF+si-*PRKN*) stained with PicoGreen (dsDNA) and MitoTracker (Mitochondria), showing cytosolic mtDNA accumulation following *PRKN* knockdown. (B) qPCR analysis of cytosolic *MT-ND1* levels in control NFs and *PRKN*-silenced NFs. (**C**) qPCR analysis of cytosolic *MT-ND2* levels in control NFs and *PRKN*-silenced NFs. Data are presented as mean ± SD from *n* = 3 biological replicates per group. Statistical significance was determined using two-tailed Student’s *t*-test. *** *p* < 0.001. Supplementary Figure S4: Effects of H_2_O_2_ exposure on KF viability and cGASmtDNA interaction. (A) CCK-8 analysis of KF viability following exposure to the indicated concentrations of H_2_O_2_ for 1 h. (B) Full uncropped gel for Figure 3E. (C) Quantification of mtDNA enrichment in cGAS immunoprecipitates relative to the corresponding IgG background. Data are presented as mean ± SD from *n* = 3 biological replicates per group. Statistical significance was determined using two-tailed Student’s *t*-test (C). ** *p* < 0.01, *** *p* < 0.001; Supplementary Figure S5: Full uncropped blot for Figure 3G; Supplementary Figure S6: Isolation and characterization of ADSCs. (A) Representative bright-field image of freshly isolated ADSCs at P0. (B) Representative bright-field image of cultured ADSCs at P3. (C,D) Representative images of multilineage differentiation assays. Adipogenic differentiation was confirmed by Oil Red O staining (C), and osteogenic differentiation was confirmed by Alizarin Red S staining (D). (E) Representative flow cytometric plots showing surface marker expression of ADSCs at P3. (F) Corresponding quantification of surface marker expression shown in (E). ADSCs exhibited high expression of mesenchymal stem cell markers (CD105, CD90, and CD73) and minimal expression of hematopoietic markers (CD45, CD14, CD34, CD19, and HLA-DR), consistent with the ISCT criteria; Supplementary Figure S7: Mitophagy-associated proteins and citrate synthase activity. (A) Full uncropped blot for Figure 5E. (B) Citrate synthase activity in untreated-, nMito-, and aMito-treated KFs after mitochondrial transplantation. Data are presented as mean ± SD from *n* = 3 biological replicates per group. Statistical significance was determined using one-way ANOVA followed by Tukey’s multiple-comparisons test. * *p* < 0.05, ** *p* < 0.01, *** *p* < 0.001; Supplementary Figure S8: Full uncropped blot for Figure 6B; Supplementary Figure S9: Full uncropped blot for Figure 8A; Supplementary Figure S10: Gross appearance and weight changes of keloid xenografts. (A) Representative gross photographs of harvested xenografts from the vehicle-, nMito-, and aMito-treated groups, shown from top to bottom, respectively. (B) Reduction in implant weight from baseline to harvest. Data are presented as mean ± SD, *n* = 6 mice per group. Statistical significance was determined using one-way ANOVA followed by Tukey’s multiple-comparisons test. * *p* < 0.05.

## Abbreviations

The following abbreviations are used in this manuscript:

2-DG	2-Deoxy-D-glucose
aMito	Adipose-derived stem cell-derived mitochondria
ADSCs	Adipose-derived stem cells
ATP	Adenosine triphosphate
CCK-8	Cell Counting Kit-8
cGAS	Cyclic GMP-AMP synthase
Col I	Type I collagen
DAMP	Damage-associated molecular pattern
DEGs	Differentially expressed genes
DMEM	Dulbecco’s modified Eagle’s medium
ECAR	Extracellular acidification rate
ECM	Extracellular matrix
H&E	Hematoxylin and eosin
KFs	Keloid fibroblasts
mtDNA	Mitochondrial DNA
NFs	Normal skin fibroblasts
nMito	Normal skin fibroblast-derived mitochondria
OCR	Oxygen consumption rate
OXPHOS	Oxidative phosphorylation
PINK1	PTEN-induced putative kinase 1
ROS	Reactive oxygen species
STING	Stimulator of interferon genes
TEM	Transmission electron microscopy
ΔΨm	Mitochondrial membrane potential
