# Supplementary material for: Mitochondrial Transplantation Suppresses mtDNA-cGAS/STING-Mediated Innate Immunity by Enhancing PINK1/Parkin-Dependent Mitophagy to Attenuate Keloid Fibrosis
Source: Antioxidants (Basel). 2026 Sep 4;15(9):1120. doi: 10.3390/antiox15091120 (PMC13603346; doi:10.3390/antiox15091120)
Supplement: Supplementary file 1 [file antioxidants-15-01120-s001.zip › antioxidants-4496473-supplementary.pdf]

Supplementary Table S1. Demographic and clinical characteristics of the human tissue donors

Group	ID	Age (years)	Sex	Site	Duration (years)
Keloid					
	1	43	M	Chest	10
	2	31	F	Chest	11
	3	53	F	Lower limb	10
	4	22	M	Back	7
	5	26	M	Back	4
	6	17	M	Chest	2
	7	35	F	Ear	10
	8	28	F	Chest	8
	9	21	F	Chest	6
	10	34	F	Chest	7
	11	23	M	Back	8
	12	19	M	Abdomen	4
	13	33	M	Ear	2
Normal skin					
	1	28	F	Abdomen	NA
	2	39	F	Abdomen	NA
	3	40	F	Abdomen	NA
	4	21	F	Abdomen	NA
	5	38	F	Abdomen	NA
	6	50	F	Abdomen	NA
Lipoaspirate					
	1	27	F	Abdomen	NA
	2	36	F	Abdomen	NA
	3	34	F	Abdomen	NA
	4	42	F	Abdomen	NA
	5	33	F	Abdomen	NA
	6	38	F	Abdomen	NA

Supplementary Table S2. Primer sequences

Target	Species	Primer	Sequence (5′–3′)	Amplicon	Annealing	Accession	Source
MT-ND1	*Homo sapiens*	Forward	CTCTTCGTCTGATCCGTCCT	100 bp	60 °C	NC_012920.1	Yu et al., 2020
		Reverse	TGAGGTTGCGGTCTGTTAGT				
MT-ND2	*Homo sapiens*	Forward	GTAGACAGTCCCACCCTCAC	109 bp	60 °C	NC_012920.1	Yu et al., 2020
		Reverse	TTGATCCCGTTTCGTGCAAG				
18SRRNA gDNA	*Homo sapiens*	Forward	GTAACCCGTTGAACCCCATT	151 bp	60 °C	NR_003286.2	Chang et al., 2013
		Reverse	CCATCCAATCGGTAGTAGCG				
L1ORF1 gDNA	*Homo sapiens*	Forward	AGAACGCCACAAAGATACTCCTCG	186 bp	60 °C	M80343.1	Papasotiriou et al., 2017
		Reverse	CTCTCTTCTGGCTTGTAGGGTTTCTG				

References

1. Yu, C. H.; Davidson, S.; Harapas, C. R.; Hilton, J. B.; Mlodzianoski, M. J.; Laohamonthonkul, P.; Louis, C.; Low, R. R. J.; Moecking, J.; De Nardo, D.; et al. TDP-43 Triggers Mitochondrial DNA Release via mPTP to Activate cGAS/STING in ALS. *Cell* **2020**, *183*, 636–649.e18.
2. Chang, J.; Liu, F.; Lee, M.; Wu, B.; Ting, K.; Zara, J. N.; Soo, C.; Al Hezaimi, K.; Zou, W.; Chen, X.; et al. NF-κB Inhibits Osteogenic Differentiation of Mesenchymal Stem Cells by Promoting β-catenin Degradation. *Proc Natl Acad Sci U S A*. **2013**, *110*, 9469–9474.
3. Papasotiriou I.; Pantopikou K.; Apostolou P. L1 Retrotransposon Expression in Circulating Tumor Cells. *PLoS One*. **2017**, *12*, e0171466.

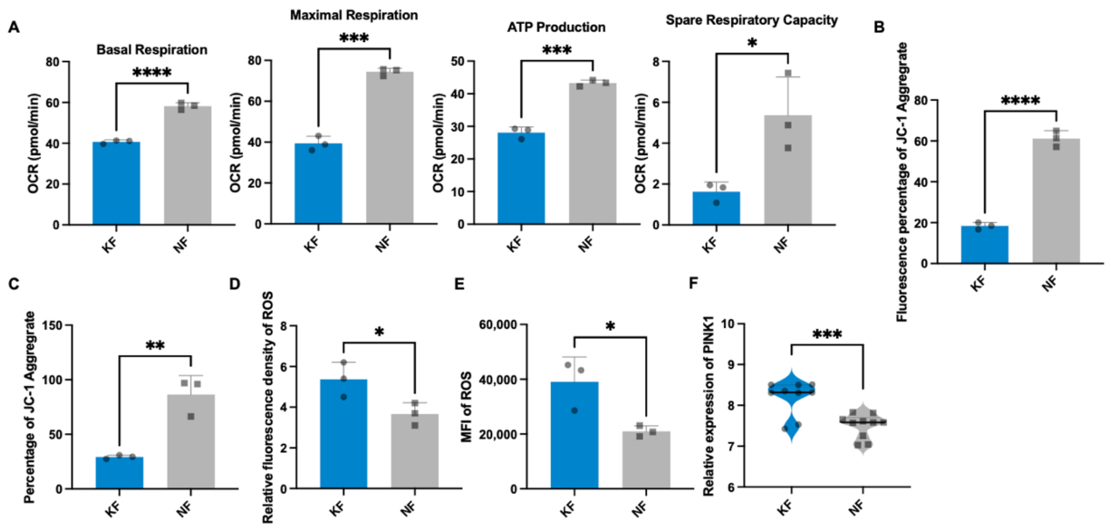

Supplementary Figure S1. Quantitative analyses of mitochondrial bioenergetics, membrane potential, oxidative stress, and bioinformatic validation in KFs. (**A**) Quantification of Seahorse mitochondrial stress test parameters, including basal respiration, maximal respiration, ATP production, and spare respiratory capacity. (**B, C**) Quantification of ΔΨm measured by JC-1 fluorescent staining using confocal microscopy (**B**) and flow cytometry (**C**). (**D, E**) Quantification of ROS levels measured by confocal microscopy (**D**) and flow cytometry (**E**), respectively. (**F**) Box plot showing relative PINK1 transcript expression in KFs versus NFs derived from the GEO dataset GSE145725. Data are presented as mean ± SD from $n = 3$ biological replicates per group. Statistical significance was determined using two-tailed Student's *t*-test. *$p < 0.05$, **$p < 0.01$, ***$p < 0.001$, ****$p < 0.0001$.

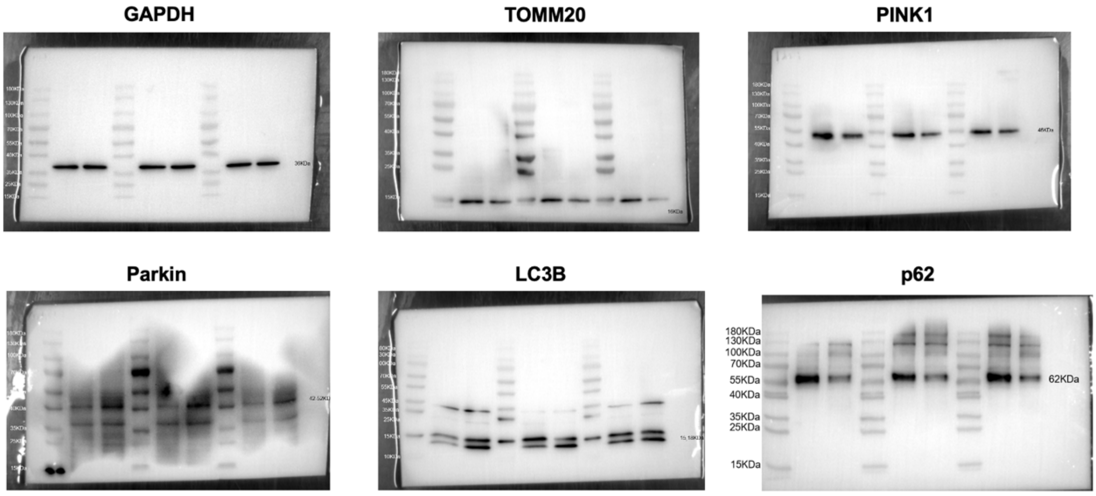

Supplementary Figure S2. Full uncropped blot for Figure 2H.

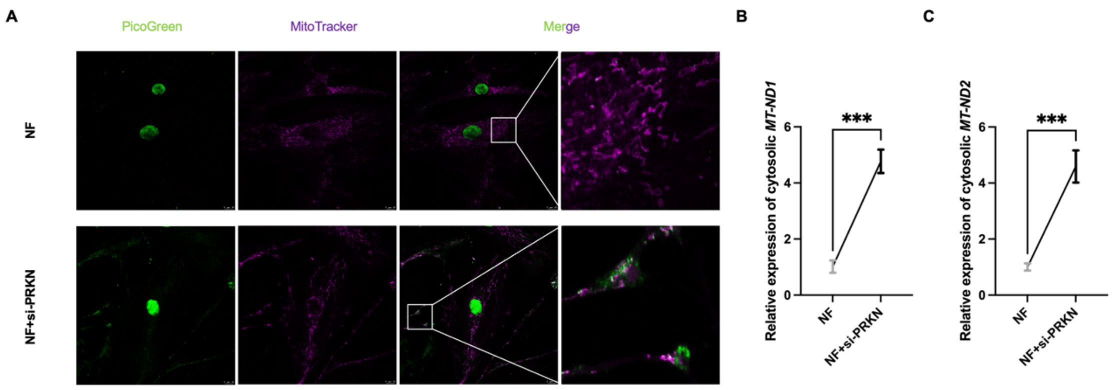

Supplementary Figure S3. Defective PINK1/Parkin-dependent mitophagy promotes mtDNA leakage. (**A**) Representative confocal microscopy images of control NFs and *PRKN*-silenced NFs (NF+si-*PRKN*) stained with PicoGreen (dsDNA) and MitoTracker (Mitochondria), showing cytosolic mtDNA accumulation following *PRKN* knockdown. (**B**) qPCR analysis of cytosolic *MT-ND1* levels in control NFs and *PRKN*-silenced NFs. (**C**) qPCR analysis of cytosolic *MT-ND2* levels in control NFs and *PRKN*-silenced NFs. Data are presented as mean ± SD from $n = 3$ biological replicates per group. Statistical significance was determined using two-tailed Student's *t*-test. ***$p < 0.001$.

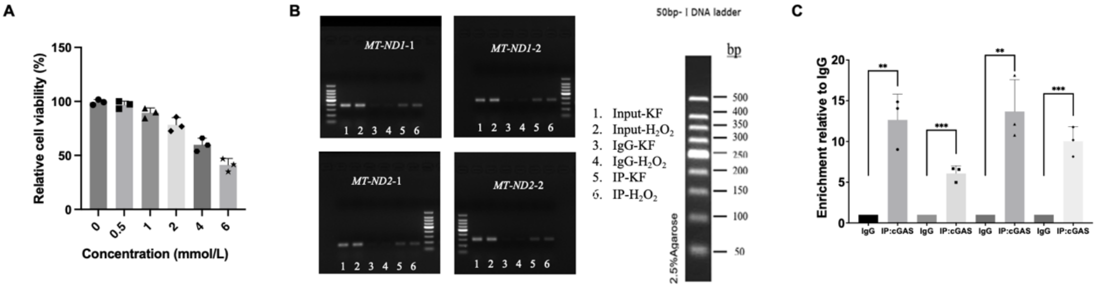

Supplementary Figure S4. Effects of H_2O_2 exposure on KF viability and cGAS-mtDNA interaction. (**A**) CCK-8 analysis of KF viability following exposure to the indicated concentrations of H_2O_2 for 1 h. (**B**) Full uncropped gel for Figure 3E. (**C**) Quantification of mtDNA enrichment in cGAS immunoprecipitates relative to the corresponding IgG background. Data are presented as mean ± SD from $n = 3$ biological replicates per group. Statistical significance was determined using two-tailed Student's *t*-test (**C**). $^{**}p < 0.01$, $^{***}p < 0.001$.

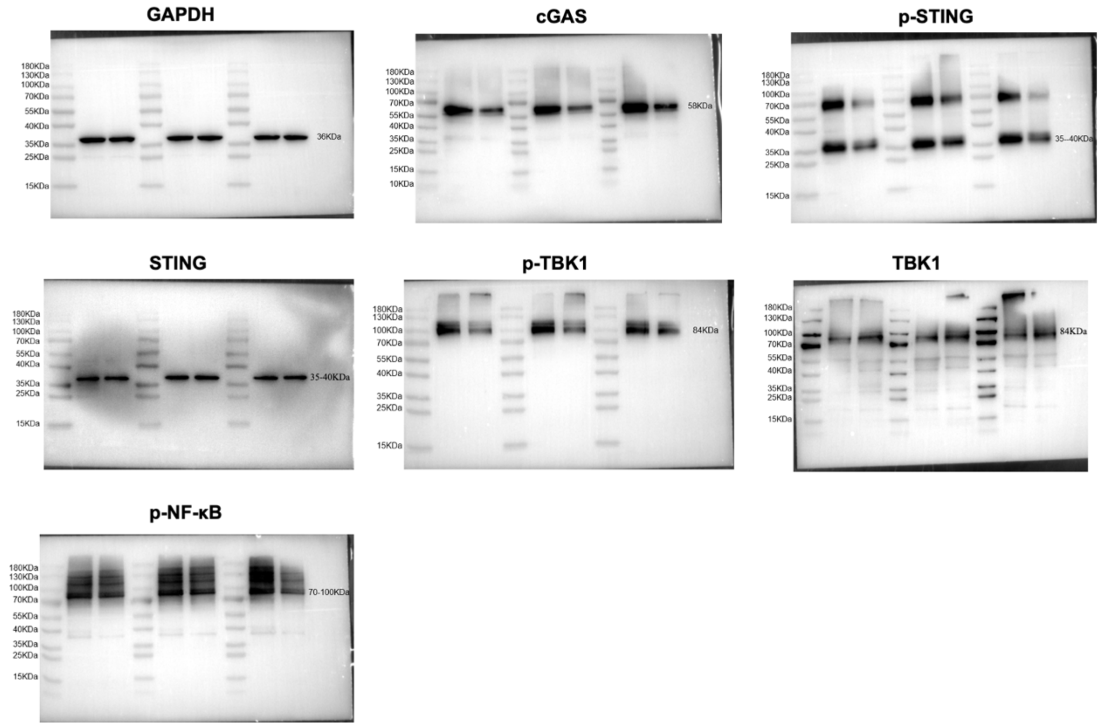

Supplementary Figure S5. Full uncropped blot for Figure 3G.

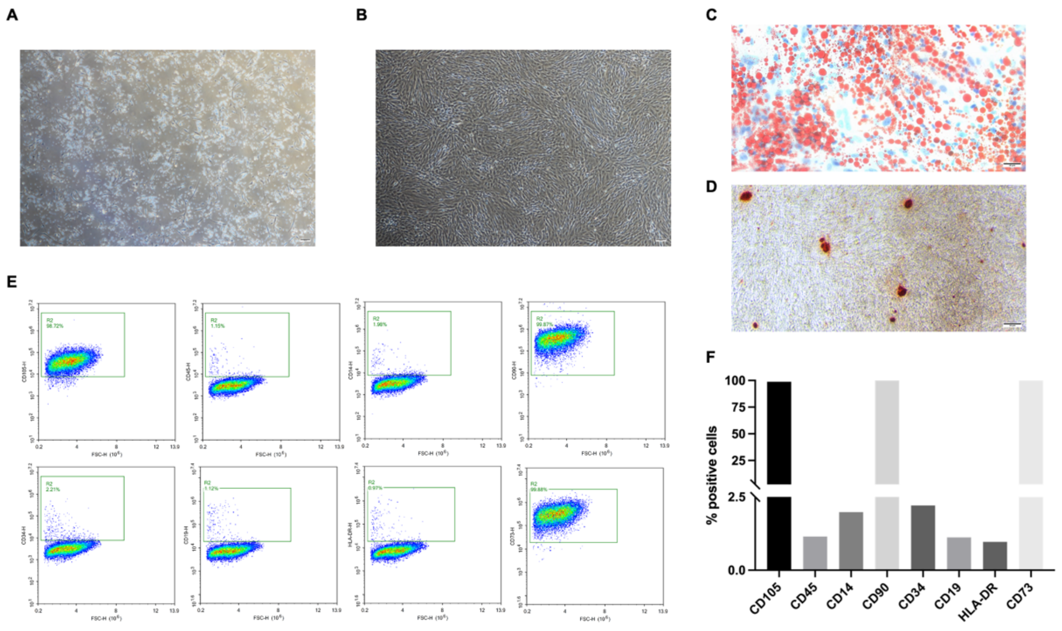

Supplementary Figure S6. Isolation and characterization of ADSCs. (**A**) Representative bright-field image of freshly isolated ADSCs at P0. (**B**) Representative bright-field image of cultured ADSCs at P3. (**C, D**) Representative images of multilineage differentiation assays. Adipogenic differentiation was confirmed by Oil Red O staining (**C**), and osteogenic differentiation was confirmed by Alizarin Red S staining (**D**). (**E**) Representative flow cytometric plots showing surface marker expression of ADSCs at P3. (**F**) Corresponding quantification of surface marker expression shown in (**E**). ADSCs exhibited high expression of mesenchymal stem cell markers (CD105, CD90, and CD73) and minimal expression of hematopoietic markers (CD45, CD14, CD34, CD19, and HLA-DR), consistent with the ISCT criteria.

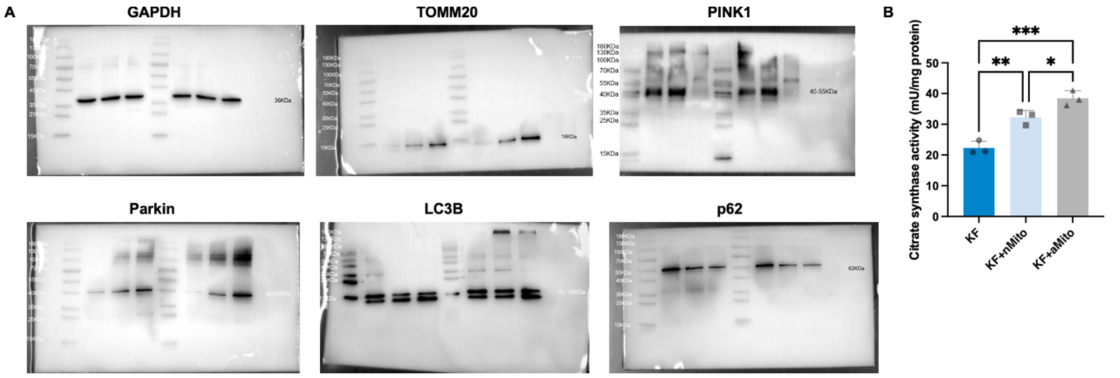

Supplementary Figure S7. Mitophagy-associated proteins and citrate synthase activity. (**A**) Full uncropped blot for Figure 5E. (**B**) Citrate synthase activity in untreated-, nMito-, and aMito-treated KFs after mitochondrial transplantation. Data are presented as mean ± SD from $n = 3$ biological replicates per group. Statistical significance was determined using one-way ANOVA followed by Tukey's multiple-comparisons test. $*p < 0.05$, $**p < 0.01$, $***p < 0.001$.

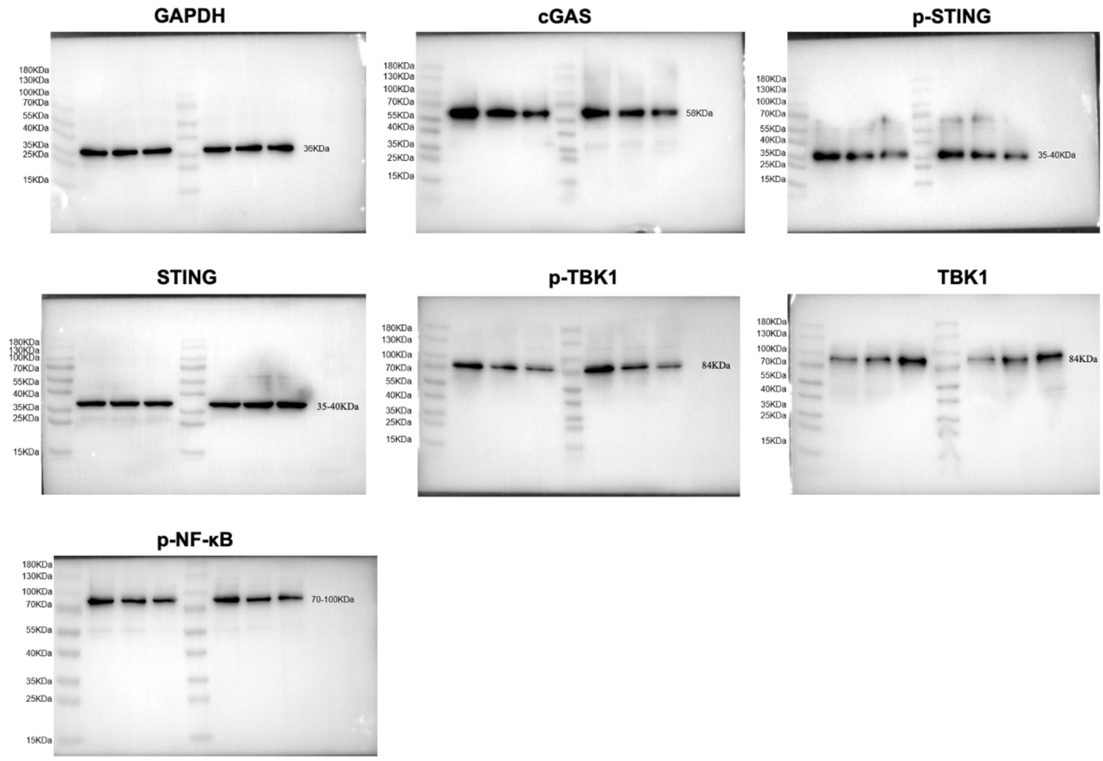

Supplementary Figure S8. Full uncropped blot for Figure 6B.

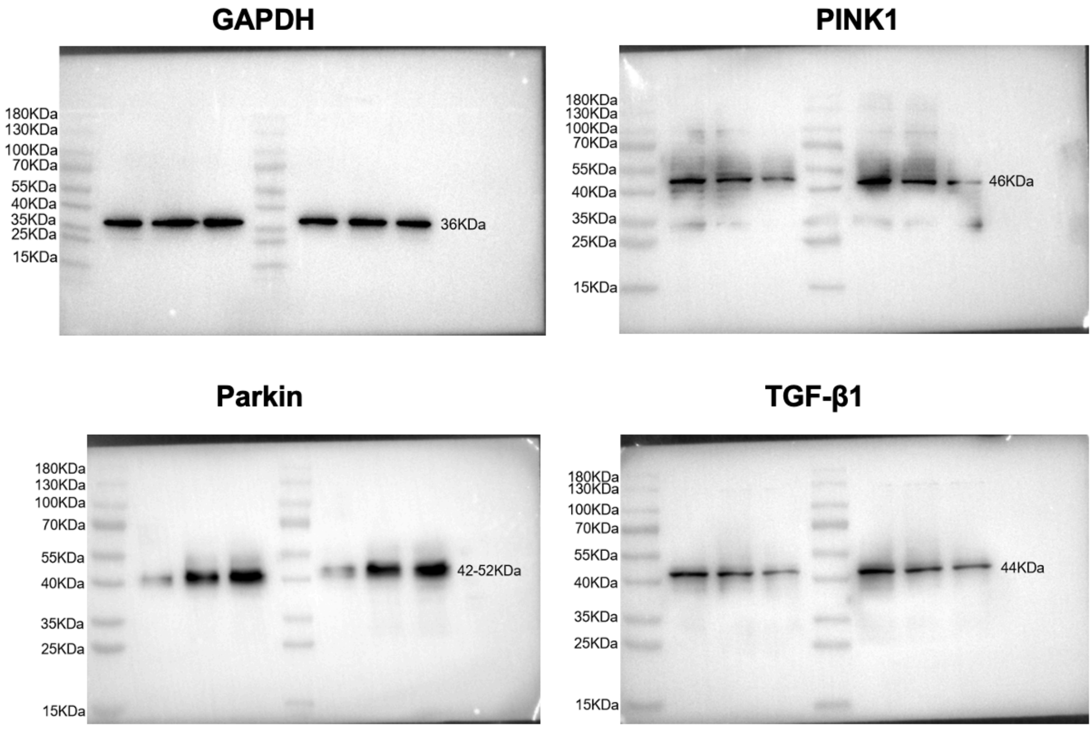

Supplementary Figure S9. Full uncropped blot for Figure 8A.

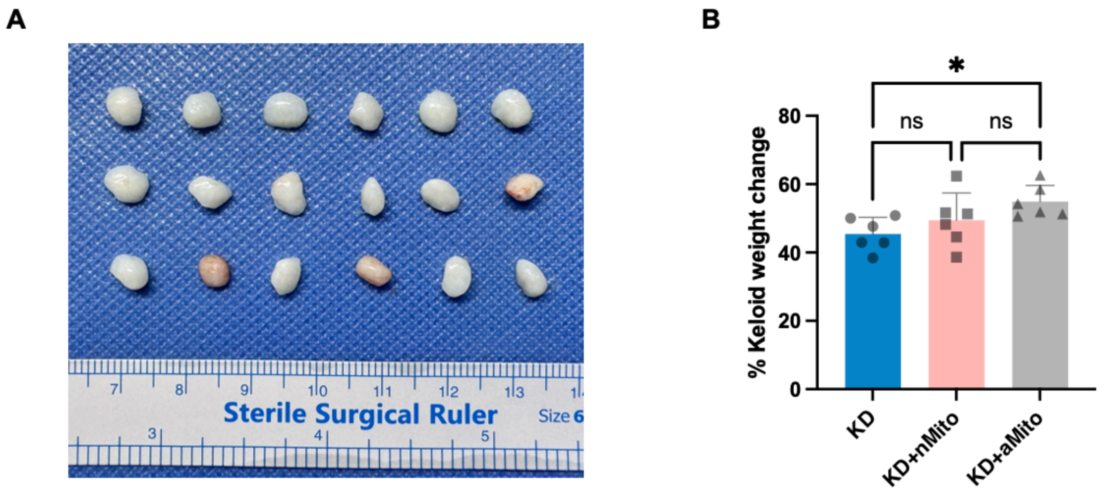

Supplementary Figure S10. Gross appearance and weight changes of keloid xenografts. (**A**) Representative gross photographs of harvested xenografts from the vehicle-, nMito-, and aMito-treated groups, shown from top to bottom, respectively. (**B**) Reduction in implant weight from baseline to harvest. Data are presented as mean ± SD, $n = 6$ mice per group. Statistical significance was determined using one-way ANOVA followed by Tukey's multiple-comparisons test. $^*p < 0.05$.
